# Contrast-Enhanced CT of the Coelom in a Female Bald Eagle (*Haliaeetus leucocephalus*): Development of a Preliminary CT-Based Anatomical Segmentation Workflow

**DOI:** 10.3390/ani16162546

**Published:** 2026-08-14

**Authors:** Alberto Arencibia, José Perera, Aday Melián, Salvador Ariza, Letizia Fiorucci, Mario Encinoso

**Affiliations:** 1 Departamento de Morfología, Facultad de Veterinaria, Universidad de Las Palmas de Gran Canaria, 35413 Arucas, Las Palmas, Spain; 2 Hospital Clínico Veterinario, Facultad de Veterinaria, Universidad de Las Palmas de Gran Canaria, 35413 Arucas, Las Palmas, Spain; jose.perera@fpct.ulpgc.es; 3 Daydream Software, 35200 Telde, Las Palmas, Spain; adaymc@gmail.com; 4 Aspro Parks Canarias SL, Palmitos Park, Barranco de los Palmitos, s/n, 35109 Maspalomas, Las Palmas, Spain; sariza@costaedutainment.com (S.A.); lfiorucci@aspro-ocio.es (L.F.)

**Keywords:** contrast-enhanced computed tomography, anatomical segmentation, avian anatomy, coelom, *Haliaeetus leucocephalus*, CT-based workflow

## Abstract

This case study describes a contrast-enhanced computed tomography (CT) examination of the coelom in a female bald eagle (*Haliaeetus leucocephalus*) and presents a structured CT-based workflow for anatomical identification and segmentation. CT images were acquired using standard imaging protocols and subsequently processed on a dedicated DICOM workstation. Multiplanar reconstructions (MPR) were generated in the transverse, sagittal, and dorsal planes to assess anatomical relationships. Maximum-intensity projection (MIP) images were generated from the MPR datasets, whereas three-dimensional volume-rendered (3D VR) images were generated directly from the CT volume. Anatomical segmentation was performed using the resulting MIP and 3D VR datasets. Anatomical structures were identified directly from the imaging data and manually segmented. The resulting segmentations were organised into a layer-based dataset to ensure consistency and traceability across the anatomical regions. This workflow provides a structured framework for the identification and organisation of anatomical structures within CT-derived datasets. An interactive visualisation environment was developed to facilitate the exploration of the segmented data, enable the dynamic display of labelled anatomical structures, and support the interpretation of the segmentation results. This study therefore presents a preliminary CT-based approach to anatomical segmentation in an avian species and may contribute to improved anatomical delineation and image interpretation in similar applications. The methodology may also support the development of analogous segmentation approaches for other avian species and related imaging applications.

## Simple Summary

This case study used contrast-enhanced computed tomography (CT) images of a female bald eagle (*Haliaeetus leucocephalus*) to develop a systematic approach for identifying and organising structures within the coelom, the main body cavity containing the visceral organs. Multiple image reconstruction techniques were applied to enhance the visualisation of the organs and associated anatomical structures. These structures were then identified, segmented, and organised into a structured anatomical dataset. An interactive visualisation tool was also developed to facilitate the exploration and interpretation of segmented structures. This preliminary workflow provides a clear and consistent representation of the bald eagle’s internal anatomy and may facilitate the interpretation of avian CT images by researchers, biologists and veterinarians. It may also serve as a valuable resource for future anatomical and diagnostic imaging studies in birds.

## 1. Introduction

The bald eagle (*Haliaeetus leucocephalus*) is a large bird of prey belonging to the family Accipitridae within the order Accipitriformes. It is widely distributed across North America, ranging from Alaska and Canada to the contiguous United States and northern Mexico. Like other members of the genus *Haliaeetus*, the species is closely associated with aquatic ecosystems and relies on both marine and freshwater habitats for foraging and breeding [1,2,3,4]. Although bald eagle (*Haliaeetus leucocephalus*) populations declined markedly during the twentieth century because of habitat loss, persecution, and exposure to organochlorine pesticides, particularly dichlorodiphenyltrichloroethane (DDT), conservation measures and legal protection have led to a substantial population recovery. Consequently, the species is currently classified as Least Concern on the IUCN Red List [2,4,5,6].

The bald eagle (*Haliaeetus leucocephalus*) exhibits pronounced sexual size dimorphism, a long lifespan, strong pair bonds and high nest-site fidelity [7,8]. These ecological and behavioural characteristics are complemented by specialised anatomical adaptations, particularly in the organisation of the avian coelom. The avian coelom exhibits a highly specialised anatomical organisation in which the visceral organs are arranged within multiple interconnected anatomical compartments. Rather than constituting a single continuous cavity surrounding the viscera, it is divided into distinct anatomical compartments that accommodate and support the major internal organs. These comprise two pulmonary pleural cavities, a single pericardial cavity, four hepatic peritoneal compartments formed by right and left divisions, each with dorsal and ventral subdivisions, and a common intestinal peritoneal cavity [9,10,11]. In addition, some organs, including the kidneys and adrenal glands, are in a retroperitoneal position and are therefore not contained within the peritoneal compartments of the coelom. This complex compartmental arrangement reflects the anatomical and physiological specialisations required to support the high metabolic demands of flight and should be considered when performing anatomical evaluations or interpreting cross-sectional imaging studies [1,4]. These characteristics make the bald eagle (*Haliaeetus leucocephalus*) a valuable species for comparative studies of avian anatomy and wildlife medicine. Despite the availability of anatomical descriptions, high-resolution characterisation of its internal structures remains limited, particularly through the application of advanced imaging and digital reconstruction techniques. Moreover, bald eagles (*Haliaeetus leucocephalus*) are affected by a wide range of traumatic, infectious, cardiovascular, parasitic, toxicological, and systemic conditions, underscoring their importance in wildlife health surveillance and rehabilitation medicine [12,13,14,15,16,17,18,19,20,21,22,23,24,25,26,27,28,29].

Computed tomography (CT) is an important imaging modality for the investigation of avian anatomy and pathology owing to its high spatial resolution and non-invasive nature [30,31]. In birds, CT is particularly valuable because of the pneumatised skeleton and the compact arrangement of the visceral organs within the coelom [32]. Contrast-enhanced CT further enhances its diagnostic utility by enabling detailed assessment of vascular structures and soft tissue [33,34]. In raptors, CT has been widely used in musculoskeletal, neurological, and forensic investigations and has consistently demonstrated superior diagnostic performance compared with conventional radiography [35,36,37,38]. Post-processing techniques, including maximum intensity projection (MIP) and three-dimensional volume rendering (3D VR), facilitate the interpretation of CT datasets by improving the visualisation of the spatial relationships among osseous, visceral, and vascular structures [39,40,41]. In avian anatomical studies, these techniques should be regarded as complementary to the interpretation of the original CT image rather than as standalone analytical methods [42,43,44].

Image segmentation is a fundamental component of medical image analysis, enabling anatomical structures to be delineated based on differences in image intensity, morphology, and spatial relationships. Manual segmentation is widely regarded as the reference standard and remains essential for the validation of computational and artificial intelligence-based methods [45]. However, despite its established role in human medicine, automated segmentation remains underutilised in veterinary diagnostic imaging, although its application is increasing, particularly in neuroimaging [46,47,48] and musculoskeletal imaging [49,50,51]. In veterinary anatomical research, manual layer-by-layer segmentation has proven valuable for generating high-fidelity reference datasets and ensuring methodological consistency and reproducibility [52,53,54,55].

In avian imaging research, computational segmentation has focused primarily on cranial anatomy, particularly using CT angiography datasets from species such as the common buzzard (*Buteo buteo*) [33]. Other applications have included 3D analysis of air sac morphology and automated segmentation during embryonic development, highlighting the potential of computational imaging techniques for anatomical reconstruction [44,56]. However, to the best of authors’ knowledge, no structured workflow for manual CT-based segmentation in the bald eagle (*Haliaeetus leucocephalus*) coelom has yet been reported.

This methodological study aimed to develop and evaluate a preliminary CT-based workflow for the anatomical identification and segmentation of the coelom in a female bald eagle (*Haliaeetus leucocephalus*) using contrast-enhanced CT. The workflow comprised the generation of MIP and 3D VR images, followed by manual layer-by-layer segmentation of the resulting datasets and the development of an interactive visualisation environment. This approach was designed to facilitate the exploration of the segmented anatomy and to provide a detailed anatomical characterisation of the coelom.

## 2. Materials and Methods

### 2.1. Animals

A 20-year-old female bald eagle (*Haliaeetus leucocephalus*) weighing 5.5 kg and housed at Aspro Parks S.L. (Palmitos Park, Maspalomas, Gran Canaria, Spain), was included in the study. The study was conducted with the owner’s consent and in accordance with applicable local animal welfare regulations. All diagnostic procedures were performed under the supervision of qualified veterinary personnel to ensure the welfare and safety of the animal throughout the examination.

### 2.2. Anaesthesia and Physiological Monitoring

The bald eagle (*Haliaeetus leucocephalus*) was anaesthetised in an induction chamber (80 cm × 60 cm × 60 cm) using 4% sevoflurane (Sevoflo, Abbott Laboratories SA, Madrid, Spain) delivered in oxygen. Chamber induction was selected to minimise handling and stress during the initial stage of anaesthesia and to facilitate the safe induction of this large raptor. Anaesthesia was subsequently maintained with 2% sevoflurane in oxygen at a flow rate of 2.0 L/min administered via a face mask. A small piece of gauze was placed over the thorax to facilitate visual monitoring of respiratory movements during spontaneous ventilation. Physiological parameters, including heart rate, respiratory rate, and oxygen saturation, were continuously monitored by pulse oximetry and clinical observation. Conventional warming blankets were used, and the ambient temperature was increased from 20 °C to 25 °C to minimise heat loss and maintain thermal stability.

### 2.3. Contrast Administration and CT Image Acquisition

A non-ionic iodinated contrast agent (iobitridol; Xenetix^®^, 300 mg I/mL; Guerbet, Villepinte, France) was administered into the left brachial vein through a 22-gauge catheter at a dose of 400 mg I/kg to enhance the visualisation of the visceral organs and vascular structures. The contrast medium was administered under veterinary supervision in accordance with standard clinical protocols to ensure the animal’s safety and physiological stability throughout the anaesthetic procedure.

Contrast-enhanced CT imaging was performed using a 16-slice helical CT scanner (Toshiba Astelion; Canon Medical Systems, Tokyo, Japan). The bald eagle (*Haliaeetus leucocephalus*) was positioned in dorsal recumbency and aligned along the craniocaudal axis. Image acquisition was performed using the following parameters: tube voltage, 100 kVp; tube current–time product, 50 mAs; slice thickness, 1 mm; reconstruction interval, 1 mm; matrix, 512 × 512; helical pitch, 0.93; and rotation time, 0.75 s.

Following imaging, the eagle was transferred to a temperature-controlled recovery enclosure and monitored until it had fully recovered from anaesthesia and resumed normal behaviour.

### 2.4. CT Image Processing

The raw CT datasets were archived and transferred to a dedicated DICOM workstation (OsiriX MD, version 13.0.2; Pixmeo, Bernex, Switzerland) for post-processing and anatomical assessment. Multiplanar reconstructions (MPR) were generated in the transverse, sagittal, and dorsal planes using OsiriX MD, version 13.0.2. MIP images were then reconstructed from the MPR to facilitate visualisation of the contrast-enhanced vasculature and other high-attenuation anatomical structures.

All images were initially reviewed using standard soft-tissue window settings (window level, 70 HU; window width, 500 HU) and lung window settings (window level, −400 HU; window width, 1500 HU). Minor adjustments to the window level and width were made as required to optimise visualisation of the coelom structures. 3D VR reconstructions were generated from the CT dataset following the manual removal of osseous structures and other non-target tissues to improve visualisation of the coelom. All CT-derived images were documented under standardised viewing conditions. The images selected for segmentation were exported from OsiriX in PNG format. Before import into the segmentation software, the exported images were processed using image-editing software (Adobe^®^ Photoshop^®^ CS5; Adobe Systems Inc., San Jose, CA, USA) solely to create a uniform black background and remove artefacts outside the anatomical region of interest. No smoothing, sharpening, contrast adjustment or modification of anatomically relevant internal boundaries was performed.

### 2.5. Imaging Data Segmentation and Interactive Visualisation Workflow

Selected CT-derived images were manually segmented by an experienced anatomist using a custom browser-based software environment developed by Daydream Software (Telde, Spain) for image annotation, data organisation and interactive visualisation. The segmentation workflow was standardised and documented to ensure procedural reproducibility and facilitate replication of the image-processing and annotation procedures using comparable datasets and software tools.

Segmentation was performed on selected MIP images reconstructed from the MPR and on selected 3D-VR views, rather than directly on the original CT volume. Throughout the segmentation process, the original transverse CT images and corresponding MPR were used as anatomical references to verify structural continuity, spatial relationships, and anatomical orientation.

Anatomical structures within the coelom were identified and labelled according to their characteristic attenuation, contrast-enhancement patterns, morphology, and spatial relationships, with reference to established veterinary anatomy textbooks [9,10,11,57]. The custom software enabled each anatomical structure to be delineated, edited, and displayed interactively as a separate layer.

#### 2.5.1. Image Organisation and Layer Management

A total of 65 CT-derived images were selected for annotation and inclusion in the interactive visualisation platform. These comprised MIP images reconstructed from the MPR and selected 3D-VR views, which provided complementary information on the morphology and spatial relationships of the coelom structures.

For each selected image, a standardised image package was created comprising raster image files at two resolutions: 1024 × 1024 pixels and 256 × 256 pixels. Individual anatomical structures were encoded as separate layers, each assigned a unique identifier and a standardised colour. Each layer consisted of a fully opaque colour mask representing a single anatomical structure on a transparent background, allowing multiple structures to be superimposed and visualised interactively.

File names contained no special characters and used underscores to maximise compatibility across operating systems and web environments. The associated metadata included image identifiers, zoom parameters and descriptive annotations, while a JSON-based label file mapped each anatomical structure to its corresponding layer identifier.

**Figure 1:**
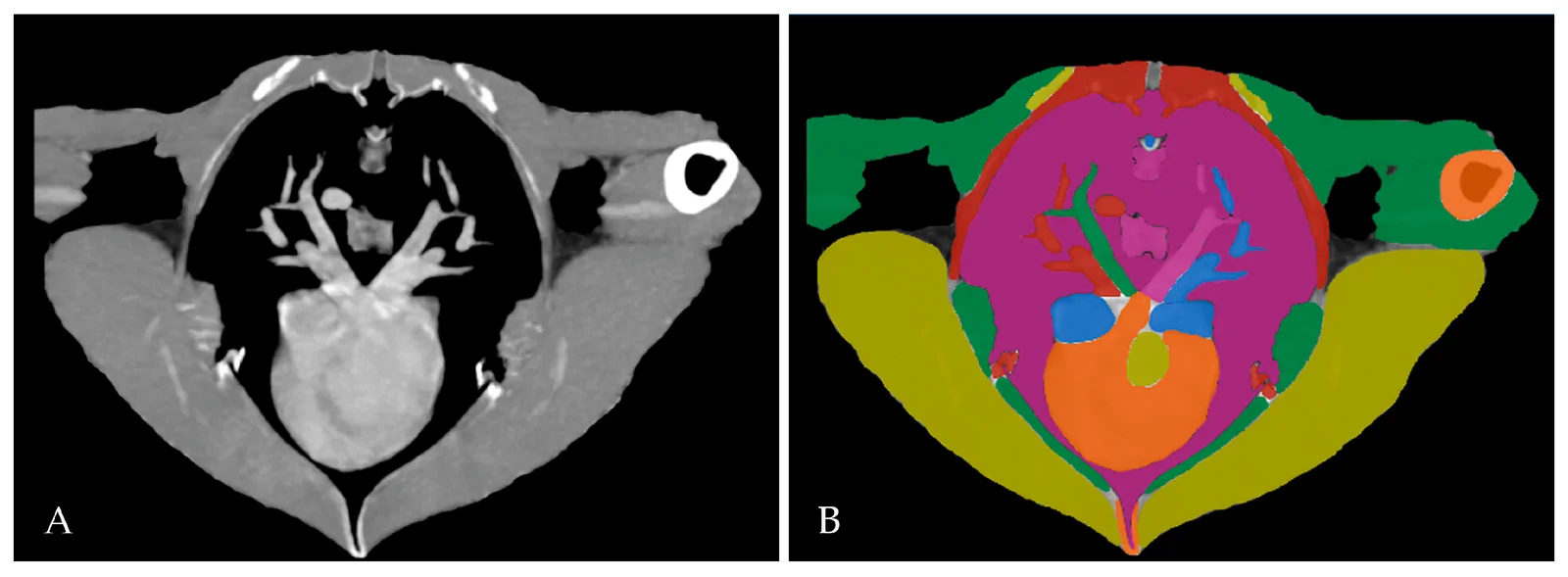
An illustrative example of layer-based manual segmentation is shown: (**A**) high-resolution source image; (**B**) corresponding segmentation overlay, different colours represent individually segmented anatomical structures.

All images and associated files were organised within a main directory comprising numbered subfolders, each corresponding to a single image. Each subfolder contained the high-resolution source image, the corresponding segmentation overlay files, and an information file (Figure 1). Metadata, credits, labels, and user-interface resources were organised using a consistent hierarchical directory structure.

#### 2.5.2. Software Implementation and Interactive Layer Editing

The custom browser-based application was developed in JavaScript (developed specifically for this study) using the Konva.js framework (version 10.3.0) [58]. Konva.js provided an HTML5 canvas-based graphical interface for displaying the source images, rendering and managing annotations layers, controlling layer visibility, enabling zooming and panning, and interactively delineating and editing anatomical structures.

Additional functions, including project initialisation, metadata management, incremental saving and reloading of annotation layers, and image package import and export, were implemented within the custom JavaScript application.

### 2.6. Interactive Visualisation System

#### 2.6.1. Data Packaging and Server Deployment

The source images, segmentation overlays, label definitions, metadata, and associated information files were assembled into standardised packages for integration into the interactive visualisation system. Each package corresponded to a single image within the platform.

The completed packages were uploaded to the web server using the File Transfer Protocol (FTP). Server-side access to these resources was managed through an application programming interface (API), enabling the visualisation client to request and retrieve the resources associated with each unique image or project identifier.

#### 2.6.2. Web-Based Visualisation Environment

A WebGL client was developed using Unity (version 6.4) [59] and adapted to provide interactive, web-based visualisation of the CT-derived images and associated segmentation layers. On initialisation, the client used a unique dataset identifier to request the corresponding resources from the server and load them on demand. The application was launched by appending a dataset query parameter to the WebGL URL (e.g., ?dataset = Name. Retrieved resources were cached locally to improve performance and minimise repeated data transfers.

**Figure 2:**
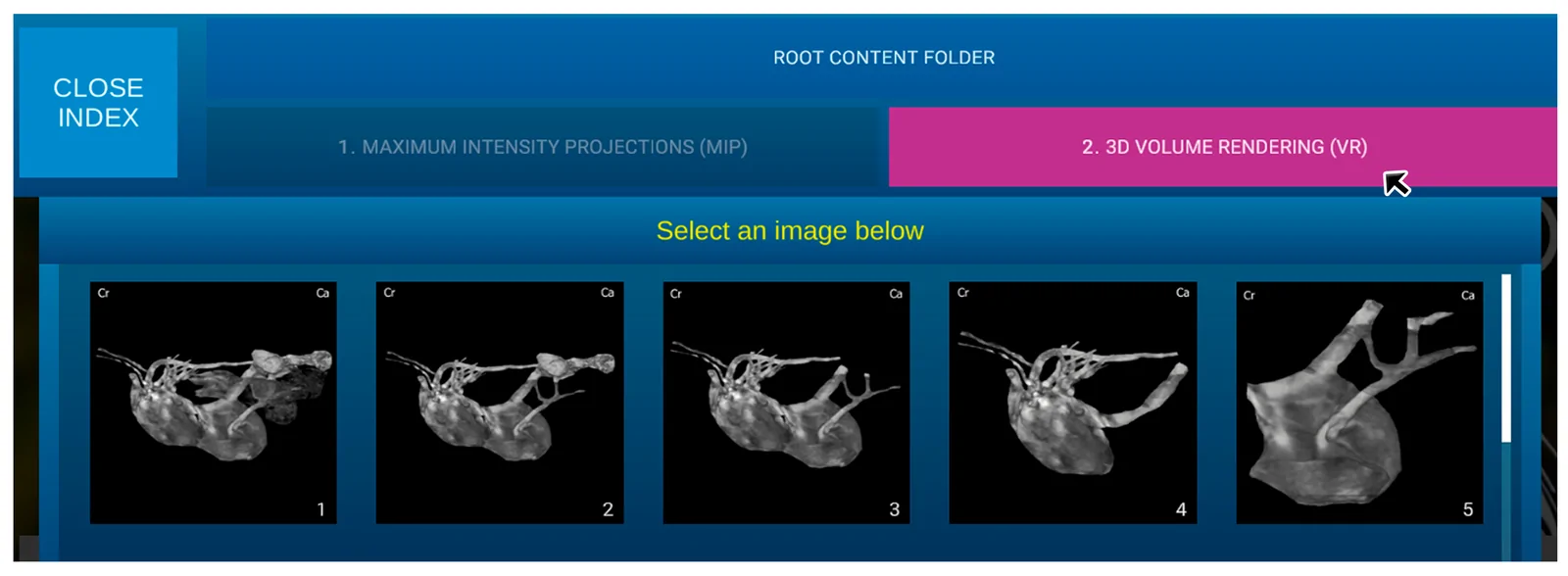
The navigation menu for all sections of the dataset is displayed. When the cursor hovers over a section (in this case, 3D VR), the section is highlighted and the corresponding images appear.

**Figure 3:**
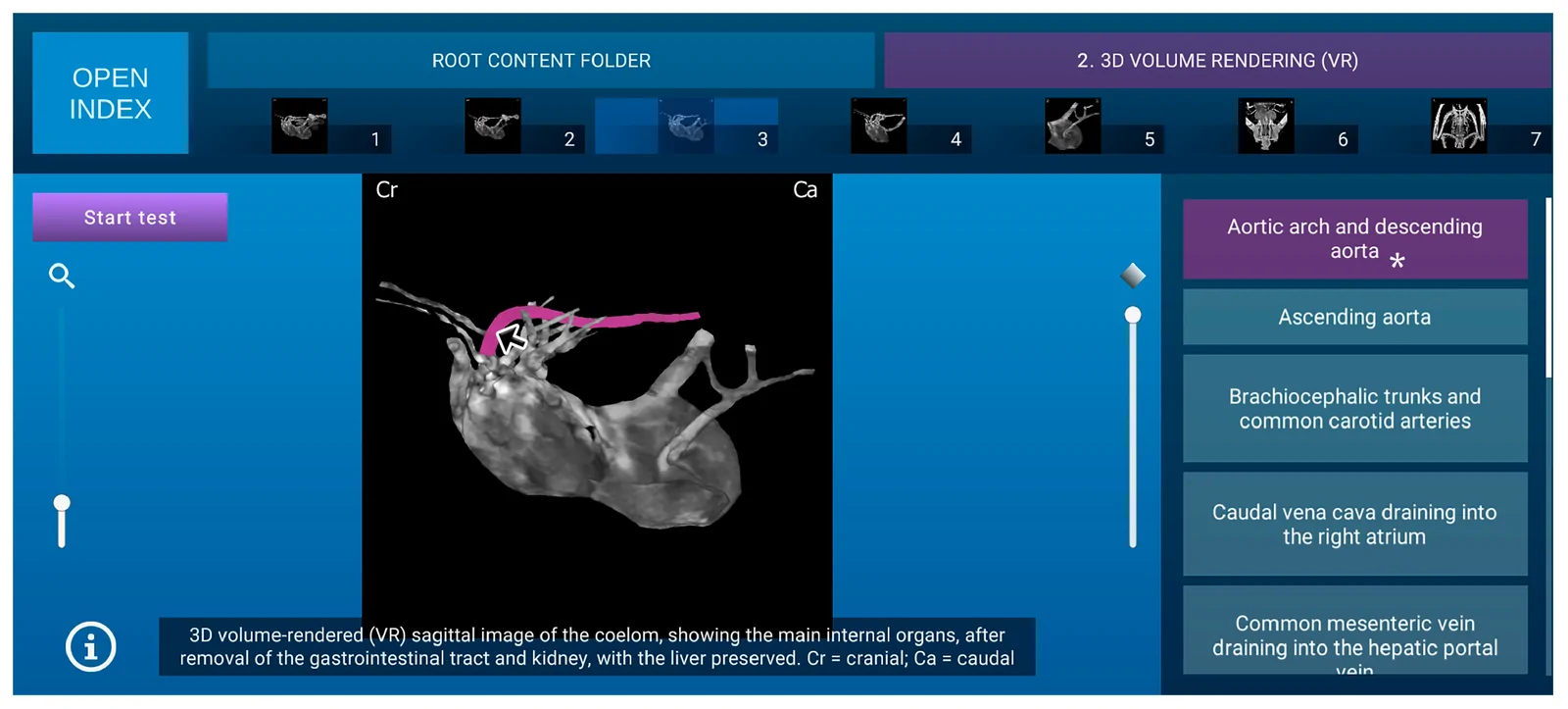
Navigation menu displaying all anatomical labels associated with a 3D VR sectional image (No. 3). When the mouse cursor is positioned over an anatomical structure in the selected image, the structure is highlighted in colour, while the corresponding label in the right-hand panel is highlighted simultaneously (white asterisk). In this example, the highlighted structures are the aortic arch and descending aorta.

**Figure 4:**
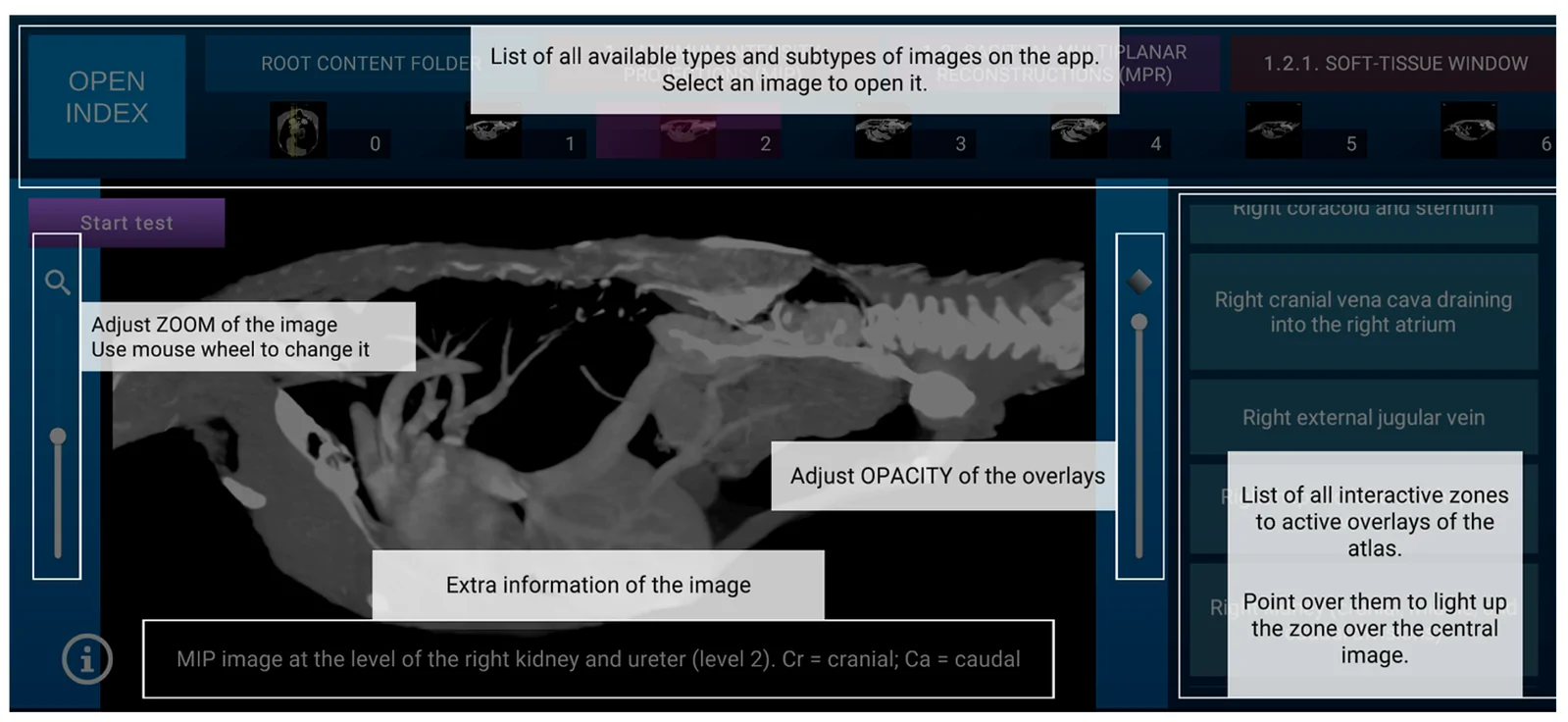
Additional resources available within the image viewer are displayed when the information icon is clicked.

The user interface comprised the following components (Figure 2, Figure 3 and Figure 4):

- an image navigation menu;
- an image viewer;
- an anatomical label panel;
- zoom and opacity controls.

The system enabled users to display, hide, and superimpose labelled anatomical structures and to interactively explore the selected MIP and 3D volume-rendered images. The platform displayed two-dimensional source images with their associated segmentation overlays but did not generate or display voxel-based 3D segmentation of the original CT volume.

This framework was developed for the interactive exploration of CT-derived anatomical images in research and educational settings. It was neither designed nor validated as a diagnostic tool or as a comprehensive anatomical atlas. Nevertheless, the resulting visualisations may provide supplementary anatomical information for research, teaching, and the interpretation of avian cross-sectional anatomy.

### 2.7. Deployment Strategy

To enable offline access, the CT-derived images and their associated segmentation files were packaged with the application and accessed via local file paths. The system was configured using the runtime parameter is Local = true, allowing the application to operate without requiring a connection to an external server.

A local file named FilesList.txt was used to define the image packages available in the directory structure. The application used this file to locate and load the corresponding source images, segmentation overlays, metadata, and associated resources directly from the local installation.

### 2.8. Self-Assessment Tool

**Figure 5:**
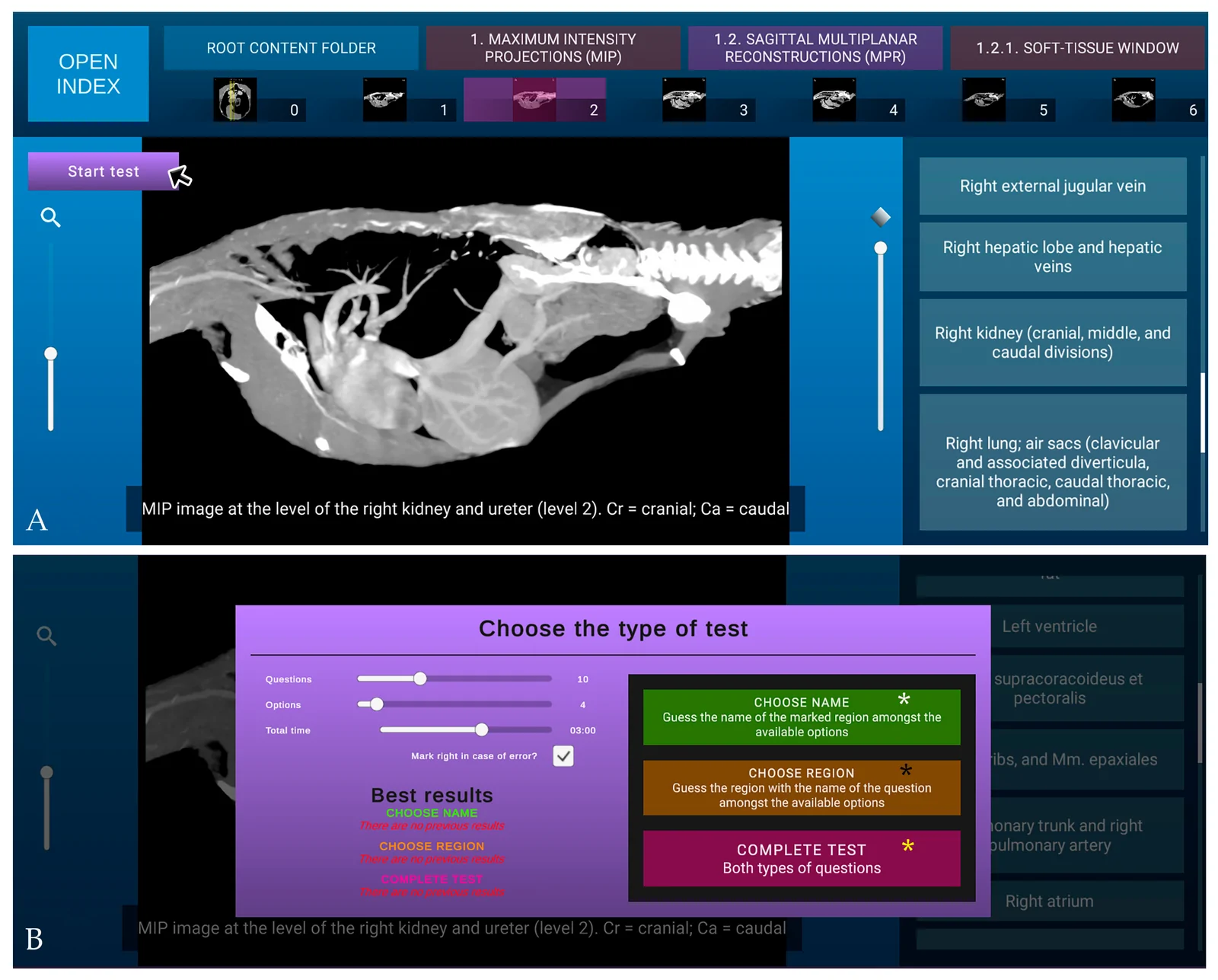
In the upper image (**A**), clicking the test interface reveals the three available assessment modalities, indicated by white, black, and yellow asterisks. The lower image (**B**) shows an expanded view of these modalities.

A self-assessment system was developed to facilitate the anatomical interpretation of CT-derived data. The system comprised three assessment modes: multiple-choice identification of anatomical structures, direct image-based selection using colour-coded overlays, and a hybrid randomised format combining both approaches. Immediate feedback was provided after each answer, and performance was scored on a scale from 0 to 10. A three-minute time limit was applied to each assessment (Figure 5).

### 2.9. Qualitative Expert Review of Anatomical Segmentation and the Interactive Visualisation Platform

During dataset development, the segmented anatomical structures, corresponding annotations, anatomical terminology, and organisation and functionality of the interactive image visualisation platform were independently reviewed by three external experts with expertise in avian anatomy, diagnostic imaging, and zoological medicine.

The review assessed the anatomical accuracy of the segmentations, the consistency of anatomical structure identification and labelling, the correct use of standardised avian anatomical terminology, and the logical organisation and functionally of the platform’s visualisation and interaction tools.

Following receipt of the experts’ feedback, the authors jointly reviewed the recommendations and reached a consensus on the revisions to be implemented. This process enabled the identification and correction of segmentation, annotation, and labelling errors; improved anatomical and terminological consistency; and refined organisation, functionality, and overall coherence of the interactive platform and the segmented datasets.

#### Data Availability

The contrast-enhanced CT imaging data are freely accessible through the following interactive visualisation platform: https://coelomiccavitybaldeagle.ulpgc.es (accessed on 30 July 2026).

## 3. Results

### 3.1. Anatomical Overview of the Coelom

MIP images and 3D VR reconstructions facilitated a comprehensive evaluation of the coelom in a female bald eagle (*Haliaeetus leucocephalus*) following intravenous contrast administration.

Although the avian coelom is anatomically organised into distinct compartments rather than a single continuous cavity surrounding the viscera, it appeared on the contrast-enhanced CT images as a continuous space containing the visceral organs together with the extensive air-sac system, with the anatomical structures distributed along the craniocaudal and dorsoventral axes. This anatomical organisation served as the anatomical framework for the topographical description of the coelom structures presented in this study.

The vertebral column, ribs and sternum provided reliable anatomical landmarks, while the body-wall musculature clearly delineated the margins of the coelom. The cardiovascular structures were located in the cranial region of the coelom. The cardiac chambers and major vessels were readily delineated. The paired cranial venae cavae and the caudal vena cava drained into the right atrium, whereas the pulmonary veins drained into the left atrium. The pulmonary trunk and pulmonary arteries were also identified. The aortic root, ascending aorta, aortic arch, descending aorta and brachiocephalic trunks were well visualised. In addition, the external jugular veins drained into the brachiocephalic veins, which in turn joined the cranial venae cavae near the thoracic inlet. Contrast enhancement enabled clear differentiation of the vascular lumina from the surrounding myocardium.

The respiratory system comprised the trachea, which bifurcated into the paired primary bronchi at the level of the syrinx, and the paired lungs, which were located dorsally within the coelom, adjacent to the vertebral column and closely associated with the dorsal surfaces of the ribs. Each lung was enclosed by the parietal pleura and covered by the visceral pleura, exhibiting the compact tubular architecture and bilateral symmetry characteristic of avian species. Owing to the rigid, non-expansile nature of the avian lungs, the pleural cavity was limited to a narrow potential space and was not discernible on the contrast-enhanced CT images.

The primary bronchi were readily identified at the pulmonary hilus, where they entered the lungs and continued into the intrapulmonary bronchial system. Although additional bronchial branches were visible within the pulmonary parenchyma, they could not be consistently distinguished according to their anatomical classification on the CT images. The air-sac system consisted of thin-walled, air-filled sacs that occupied much of the coelom and surrounded many of the visceral organs. It comprised the paired cervical, cranial thoracic, caudal thoracic, and abdominal air sacs, together with the unpaired clavicular air sac. The cervical air sacs gave rise to the vertebral diverticula, whereas the clavicular air sac gave rise to the interclavicular, sternal, subpectoral, subscapular, axillary, and humeral diverticula. The paired abdominal air sacs extended caudally within the coelom and were associated with the pelvic, perirenal, and femoral recesses. Collectively, these structures formed close anatomical relationships with the lungs, heart, major blood vessels, visceral organs, and both the axial and appendicular skeleton, contributing to the extensive skeletal pneumatisation. Although portions of the thin membranous walls separating adjacent air sacs were discernible on CT images, they could not be visualised consistently enough to permit reliable delineation and segmentation of the individual air sacs, their associated diverticula, and abdominal recesses. Consequently, the anatomical description presented in this study was confined to structures that could be identified reliably on CT.

The digestive tract followed a predominantly craniocaudal course within the coelom and was located mainly within the common intestinal peritoneal cavity, which contains most of the gastrointestinal tract. The oesophagus continued into the glandular stomach (proventriculus), which in turn communicated with the muscular stomach (ventriculus), before extending caudally into the intestinal loops. The liver was situated immediately caudal to the heart and extended bilaterally within the coelom, comprising two well-defined lobes. Although closely associated with the liver, the gallbladder was clearly visualised and readily distinguished from the surrounding hepatic parenchyma, allowing its anatomical margins to be delineated accurately. The spleen was located adjacent to the proventriculus. The pancreas was not clearly visualised, most likely because of its close anatomical relationship with the duodenal loop and the sorrounding soft tissues.

Several major vascular structures were identified within the coelom, including the coeliac and hepatic arteries, and the hepatic portal vein and its splenic and proventricular tributaries. Within the hepatic parenchyma, the hepatic veins were clearly visualised as distinct vascular structures. The cranial and caudal mesenteric veins united to form the common mesenteric vein, which drained into the hepatic portal system. The caudal vena cava was formed by the confluence of the right and left common iliac veins and coursed cranially through the coelom.

The urinary system comprised paired kidneys located retroperitoneally within the renal fossae of the synsacrum. Each kidney was clearly divided into cranial, middle, and caudal divisions and lay in close apposition to the synsacrum, reflecting its intimate anatomical relationship with the pelvic region. The adrenal glands were situated adjacent to the cranial division of each kidney. They appeared as elongated structures positioned medially and partially embedded within the connective tissue surrounding the renal parenchyma. The ureters arose from the kidneys and extended caudally along the dorsal body wall towards the cloaca. Renal and pelvic portions could be distinguished along their course, with a gradual transition between them. The pelvic portion of each ureter continued caudally and opened into the urodeum, the intermediate compartment of the cloaca.

In this species, the gonadal structures consisted of a single, relatively small left ovary located adjacent to the cranial pole of the left kidney, consistent with the typical reproductive anatomy of female birds. No right ovary was identified. The nervous system was assesed during image interpretation; however, no peripheral nerves, including the sacral and pudendal plexuses, could be identified reliably on the CT images. Consequently, these structures were excluded from the anatomical description.

### 3.2. Contrast-Enhanced CT Assessment: MIP and 3D VR Reconstructions

The contrast-enhanced CT dataset was post-processed using two complementary techniques: MIP and 3D VR reconstruction. Standard reference images preceded the MIP reconstructions to indicate the approximate anatomical level represented in each imaging plane, facilitating spatial orientation during interpretation of the reconstructed datasets. MIP reconstructions were generated in the transverse, sagittal, and dorsal planes according to the standardised protocol.

Transverse MIP reconstructions were generated sequentially in a craniocaudal direction, extending from the level of the cranial venae cavae to the caudal divisions of the kidneys. Soft-tissue and lung window settings were applied, generating 12 images per dataset. Sagittal MIP reconstructions were generated sequentially from right to left, extending from the right brachiocephalic trunk to the left brachiocephalic trunk, using the same window settings to generate six images per dataset. Dorsal MIP reconstructions were generated sequentially in a dorsoventral direction, extending from the kidneys and ureters to the cardiac ventricles and generating eight images per dataset.

Image orientation was standardised across all datasets in accordance with radiological convention. The dorsal aspect of the coelom was displayed uppermost, with the right side of the animal shown on the left side of the image. 3D VR reconstructions were generated as part of the post-processing workflow. A total of seven 3D VR images were produced, comprising five sagittal and two dorsal reconstructions. Together, the MIP and 3D VR reconstructions provided a comprehensive 3D assessment of the coelom following intravenous contrast administration, facilitating the evaluation of contrast enhancement and the delineation of anatomical structures.

#### 3.2.1. MIP Reconstructions Using a Soft-Tissue Window

Under soft-tissue window settings, air-filled spaces, soft tissues, and bone were readily distinguished. The vertebral column, ribs, sternum, and synsacrum exhibited heterogeneous attenuation. Cortical bone was markedly hyperattenuating, whereas several skeletal regions showed lower attenuation consistent with physiological pneumatisation. The surrounding musculature exhibited homogeneous intermediate attenuation, allowing clear differentiation from adjacent soft tissues.

Following intravenous contrast administration, the cardiovascular structures exhibited strong, homogeneous enhancement. The vascular lumina were markedly hyperattenuating relative to the sorrounding soft tissues, enabling clear delineation of the major arterial and venous pathways. The myocardium displayed intermediate attenuation and was readily distinguished from the contrast-opacified vessels. The air-filled components of the respiratory system were markedly hypoattenuating, providing excellent intrinsic contrast with the surrounding soft tissues and allowing clear visualisation of the distribution of the pneumatic system throughout the coelom.

The liver and spleen exhibited homogeneous soft-tissue attenuation and uniform contrast enhancement. The gallbladder was visualised as a hypoattenuating structure relative to the surrounding hepatic parenchyma, although its margins could only be partially delineated. Nevertheless, the difference in attenuation allowed reliable anatomical identification. The pancreas could not be clearly distinguished, most likely because of the limited soft tissue contrast between the pancreatic parenchyma and the adjacent gastrointestinal structures. The gastrointestinal tract exhibited intermediate mural attenuation and low-attenuation luminal contents, allowing clear differentiation between the intestinal wall and lumen.

**Figure 6:**
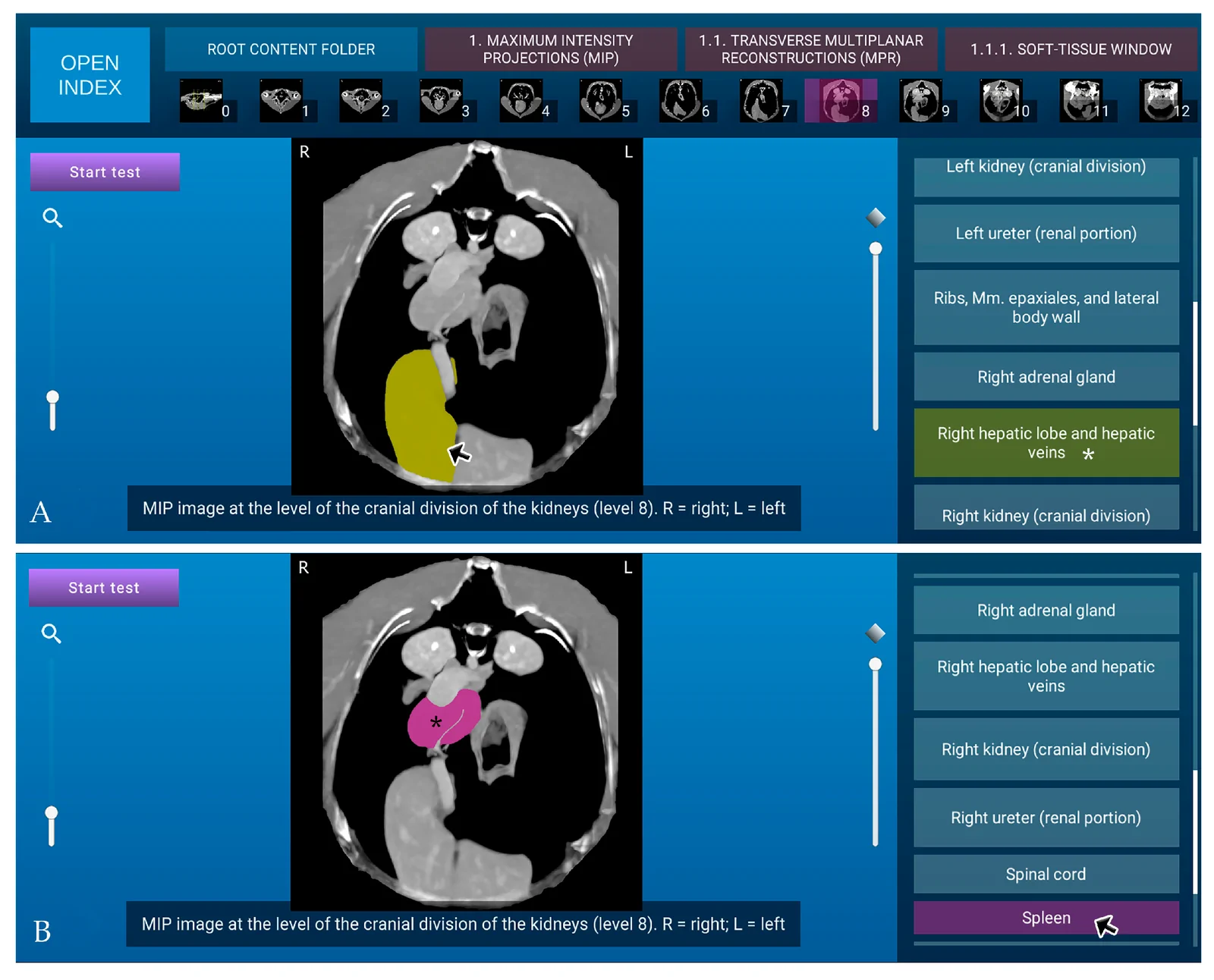
Representative transverse MIP reconstruction displayed in a soft-tissue window at the level of the cranial divisions of the kidneys (level 8), viewed cranially. (**A**) Positioning the cursor over a structure highlights it in colour and simultaneously highlights the corresponding label (right hepatic lobe and hepatic veins; white asterisk). (**B**) Positioning the cursor over a label (spleen) highlights the corresponding anatomical structure (black asterisk).

**Figure 7:**
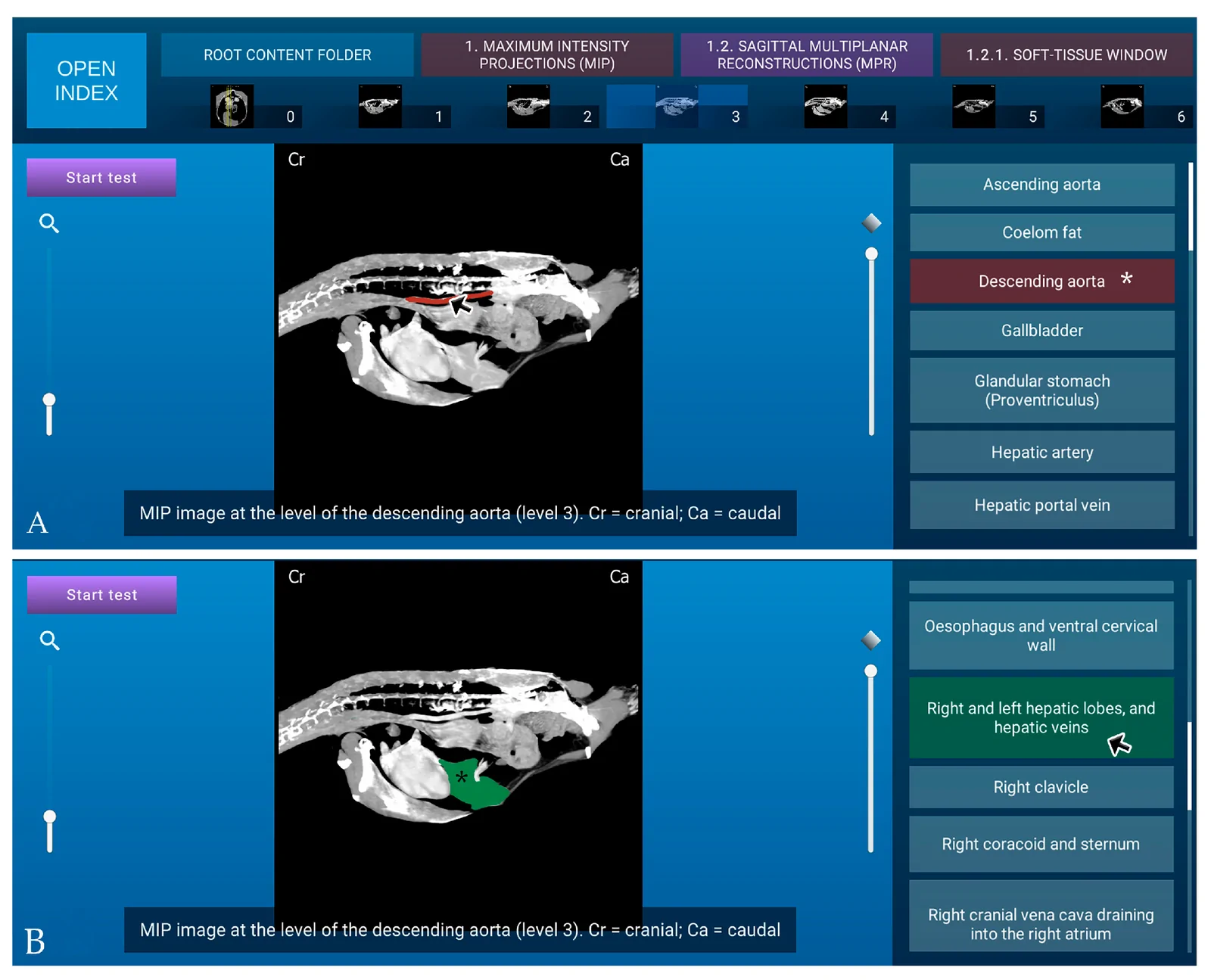
Representative sagittal MIP reconstruction displayed using a soft-tissue window at the level of the descending aorta (level 3), viewed from the left lateral side. (**A**) Hovering the cursor over a structure highlights it in colour and simultaneously highlights the corresponding label (Descending aorta; white asterisk). (**B**) Hovering the cursor over a label highlights the corresponding anatomical structure: right and left hepatic lobes, and hepatic veins (black asterisk).

**Figure 8:**
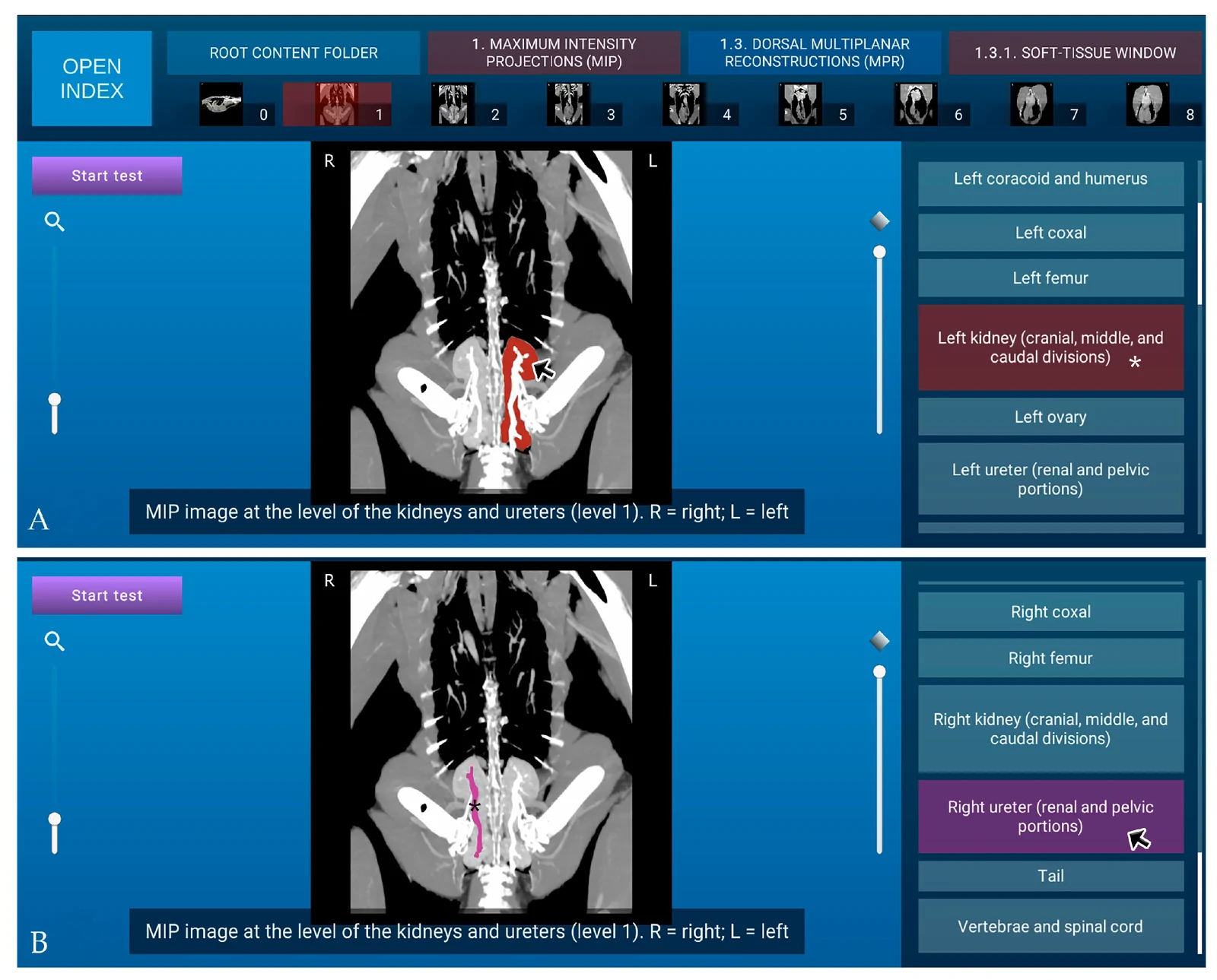
Representative dorsal MIP reconstruction displayed using a soft-tissue window at the level of the kidneys and ureters (level 1), viewed ventrally. (**A**) Hovering the cursor over a structure highlights it in colour and simultaneously highlights the corresponding label (left kidney, including the cranial, middle, and caudal divisions; white asterisk). (**B**) Hovering the cursor over a label highlights the corresponding anatomical structure (right ureter; black asterisk).

The kidneys exhibited moderate soft-tissue attenuation, with preservation of their internal architecture. The adrenal glands were faintly visualised medial to the cranial divisions of the kidneys and remained poorly differentiated from the adjacent renal parenchyma. Following intravenous contrast administration, the ureters were identified as thin, hyperattenuating tubular structures extending caudally towards the cloaca. The left ovary exhibited homogeneous soft-tissue attenuation but remained poorly differentiated from the surrounding tissues, whereas no right ovary was identified. Representative MIP reconstructions obtained using soft-tissue window settings are presented in Figure 6, Figure 7 and Figure 8.

#### 3.2.2. MIP Reconstructions Using a Lung Window

Following intravenous contrast administration, lung window settings enhanced the visualisation of air-filled structures and their spatial relationships, enabling detailed assessment of the avian respiratory system and its associated anatomical structures. The pulmonary parenchyma was markedly hypoattenuating, with well-defined margins and a finely internal architecture consistent with normal aeration. The trachea, syrinx, and primary bronchi were clearly visualised as air-filled tubular structures with hypoattenuating lumina enclosed by thin soft-tissue walls.

The primary bronchi and their intrapulmonary branches were readily visualised within the pulmonary parenchyma as air-filled tubular structures with hypoattenuating lumina enclosed by thin soft-tissue walls. The continuity of the bronchial tree was readily appreciated, facilitating assessment of airway distribution and bilateral symmetry. Owing to their markedly low attenuation, the air sacs were prominently visualised as an extensive network of thin-walled, hypoattenuating compartments occupying a substantial proportion of the coelom. The cervical, clavicular, cranial thoracic, caudal thoracic, and abdominal air sacs could be distinguished on the basis of their anatomical location and relationships with adjacent structures. However, their individual boundaries could not be delineated reliably because the thin membranous walls separating adjacent air sacs were imperceptible or only faintly visible under normal conditions.

Osseous structures were more readily appreciated on lung window settings, particularly in regions of physiological pneumatisation. The synsacrum, vertebral bodies, and sternum exhibited low-attenuation air-filled spaces within the bone, consistent with normal skeletal pneumatisation. Cortical bone remained markedly hyperattenuating, providing clear definition of the osseous margins against the adjacent aerated spaces. Soft tissues, including the musculature and coelom organs, appeared relatively hyperattenuating; however, their internal detail was reduced because the window settings were optimised for visualising of air-containing structures.

The heart exhibited homogeneous soft-tissue attenuation, with uniform contrast opacification of the cardiac chambers; however, myocardial definition was less distinct than on soft-tissue window settings. Although the major blood vessels remained readily identifiable following intravenous contrast administration, they were less conspicuous on lung window settings.

Parenchymal organs, including the liver, spleen, and kidneys, could be identified on the basis of their anatomical location and relative soft-tissue attenuation; however, their internal architecture and contrast-enhancement patterns were less clearly defined than on soft-tissue window settings. The adrenal glands were only faintly discernible because of the limited soft-tissue contrast provided by the lung window settings and remained poorly differentiated from the adjacent renal parenchyma.

The gallbladder was also poorly visualised, appearing as a faintly hypoattenuating structure with indistinct margins that blended with the surrounding hepatic parenchyma. The pancreas could not be identified because it was not reliably distinguishable from the adjacent gastrointestinal and coelom soft tissues. The gastrointestinal tract was variably delineated, with intraluminal gas improving visualisation of individual segments in some regions. The left ovary was only faintly discernible, whereas no right ovary was identified.

**Figure 9:**
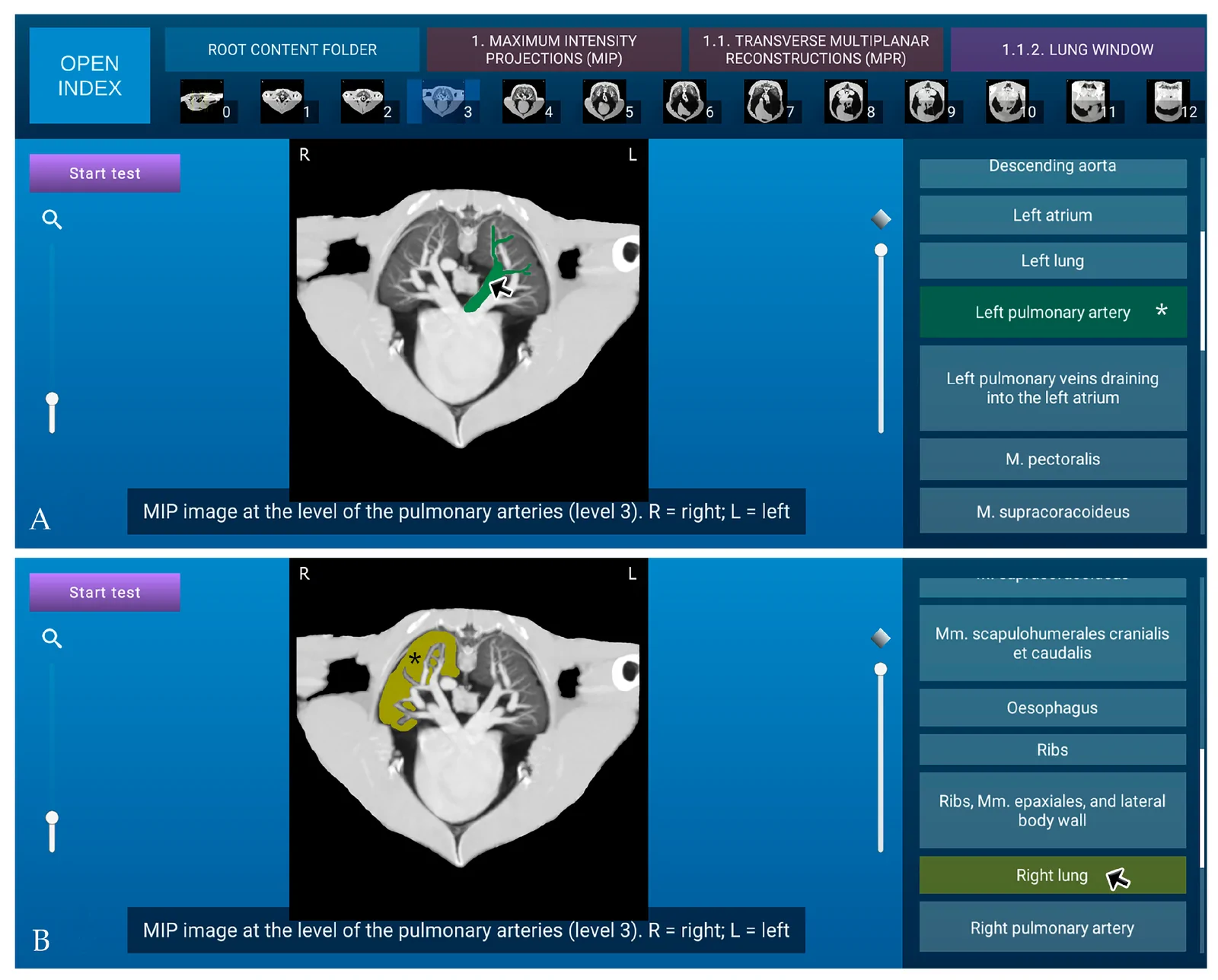
Representative transverse MIP reconstruction displayed using a lung window at the level of the pulmonary arteries (level 3), viewed cranially. (**A**) Hovering the cursor over a structure highlights it in colour and simultaneously highlights the corresponding label (left pulmonary artery; white asterisk). (**B**) Hovering the cursor over a label highlights the corresponding anatomical structure (right lung; black asterisk).

**Figure 10:**
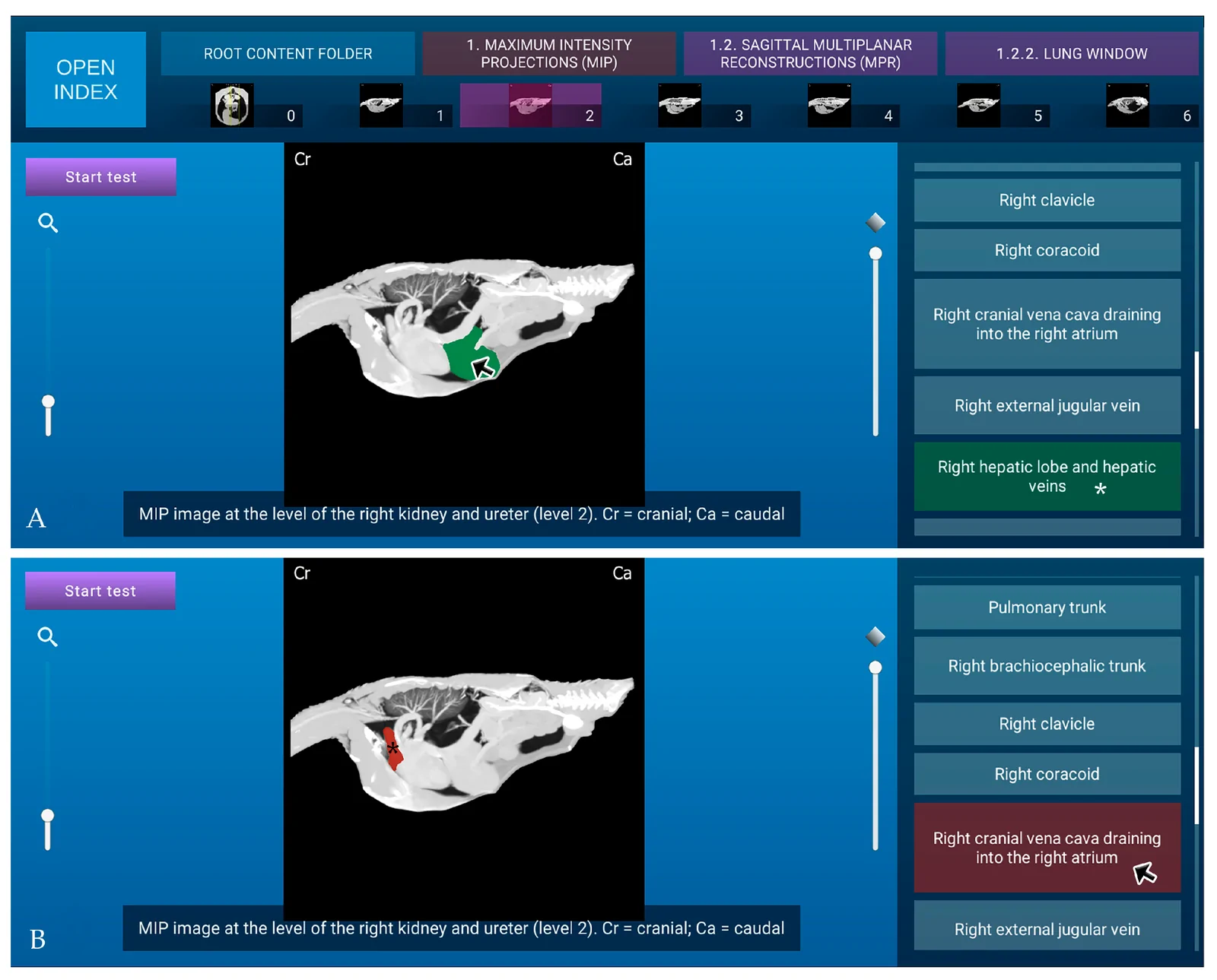
Representative sagittal MIP image displayed in a lung window at the level of the right kidney and ureter (level 2), viewed from the left lateral aspect. (**A**) Positioning the cursor over a structure highlights it in colour and simultaneously highlights the corresponding label (right hepatic lobe and hepatic veins; white asterisk). (**B**) Positioning the cursor over a label (right cranial vena cava draining into the right atrium) highlights the corresponding anatomical structure (black asterisk).

**Figure 11:**
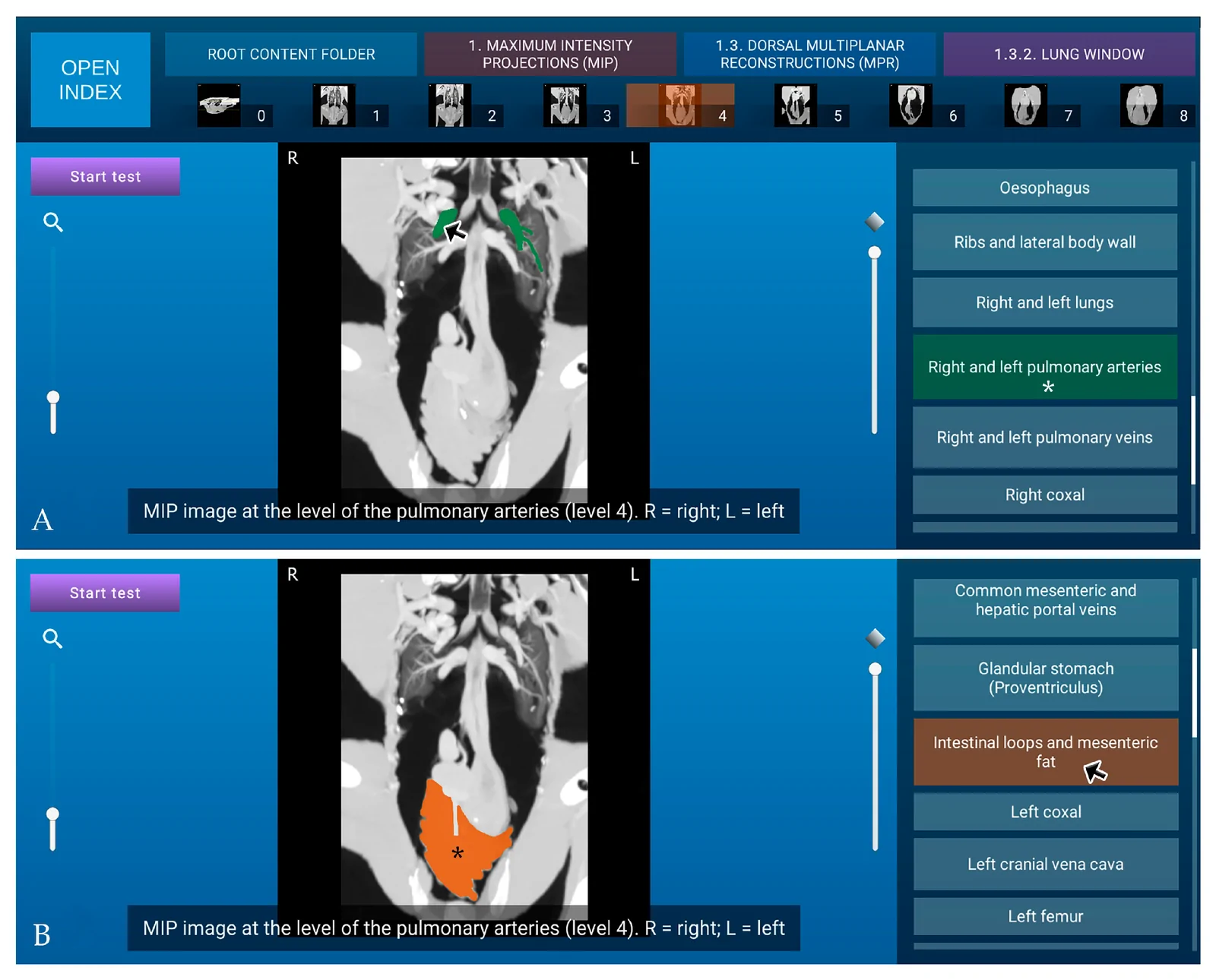
Representative dorsal MIP reconstruction displayed in a lung window at the level of the pulmonary arteries (level 4), viewed from the ventral aspect. (**A**) Positioning the cursor over a structure highlights it in colour and simultaneously highlights the corresponding label (right and left pulmonary arteries; white asterisk). (**B**) Positioning the cursor over a label (intestinal loops and mesenteric fat) highlights the corresponding anatomical structure (black asterisk).

Overall, MIP reconstructions generated using lung window settings highlighted the extent and spatial organisation of the avian pneumatic system. Compared with soft-tissue window settings, they provided enhanced visualisation of aerated structures while complementing the anatomical information obtained from soft-tissue reconstructions. Representative lung window MIP reconstructions are presented in Figure 9, Figure 10 and Figure 11.

#### 3.2.3. 3D VR Reconstructions

3D VR reconstructions in sagittal and dorsal orientations provided semi-transparent visualisation of the coelom structures, enabling simultaneous assessment of the organs and skeletal elements within their 3D anatomical context. This approach enabled integrated assessment of the spatial relationships and overall anatomical organisation of the coelom.

Attenuation-based VR was instrumental in tissue differentiation, with variations in voxel attenuation translated into corresponding differences in opacity. Highly attenuating structures, including cortical bone and contrast-opacified vascular lumina, were displayed with greater opacity, making them readily distinguishable. By contrast, soft tissues with intermediate attenuation values were assigned partial opacity, enabling layered visualisation of overlapping anatomical structures.

Targeted VR reconstructions of selected coelom structures were generated by adjusting opacity thresholds and selecting specific attenuation ranges. This approach enhanced visualisation of the cardiovascular system, including the cardiac chambers and major blood vessels, which were clearly delineated as a result of their marked post-contrast enhancement. Consequently, their spatial orientation and relationships within the coelom could therefore be readily assessed.

Solid organs, including the liver, spleen, and kidneys, were clearly visualised, enabling assessment of their morphology, relative position, and anatomical relationships. The gastrointestinal tract exhibited intermediate attenuation and could be rendered with adjustable transparency, allowing evaluation in the context of the surrounding anatomical structures.

Osseous structures exhibited heterogeneous attenuation, with hyperattenuating cortical margins surrounding internal regions of lower attenuation that reflected physiological skeletal pneumatisation. This attenuation pattern enabled simultaneous assessment of the skeletal architecture and adjacent soft tissues, providing a comprehensive representation of the 3D organisation of the avian skeleton.

In contrast, the pulmonary parenchyma and air sacs were not adequately visualised on the VR reconstructions. Their very low attenuation rendered them almost completely transparent under the selected rendering parameters, markedly reducing their conspicuity and precluding detailed assessment of the avian pneumatic system.

VR post-processing enabled interactive manipulation of the viewing planes, real-time adjustment of opacity thresholds, and selective segmentation of anatomical structures. These capabilities facilitated targeted visualisation and improved interpretation of complex 3D anatomical relationships.

**Figure 12:**
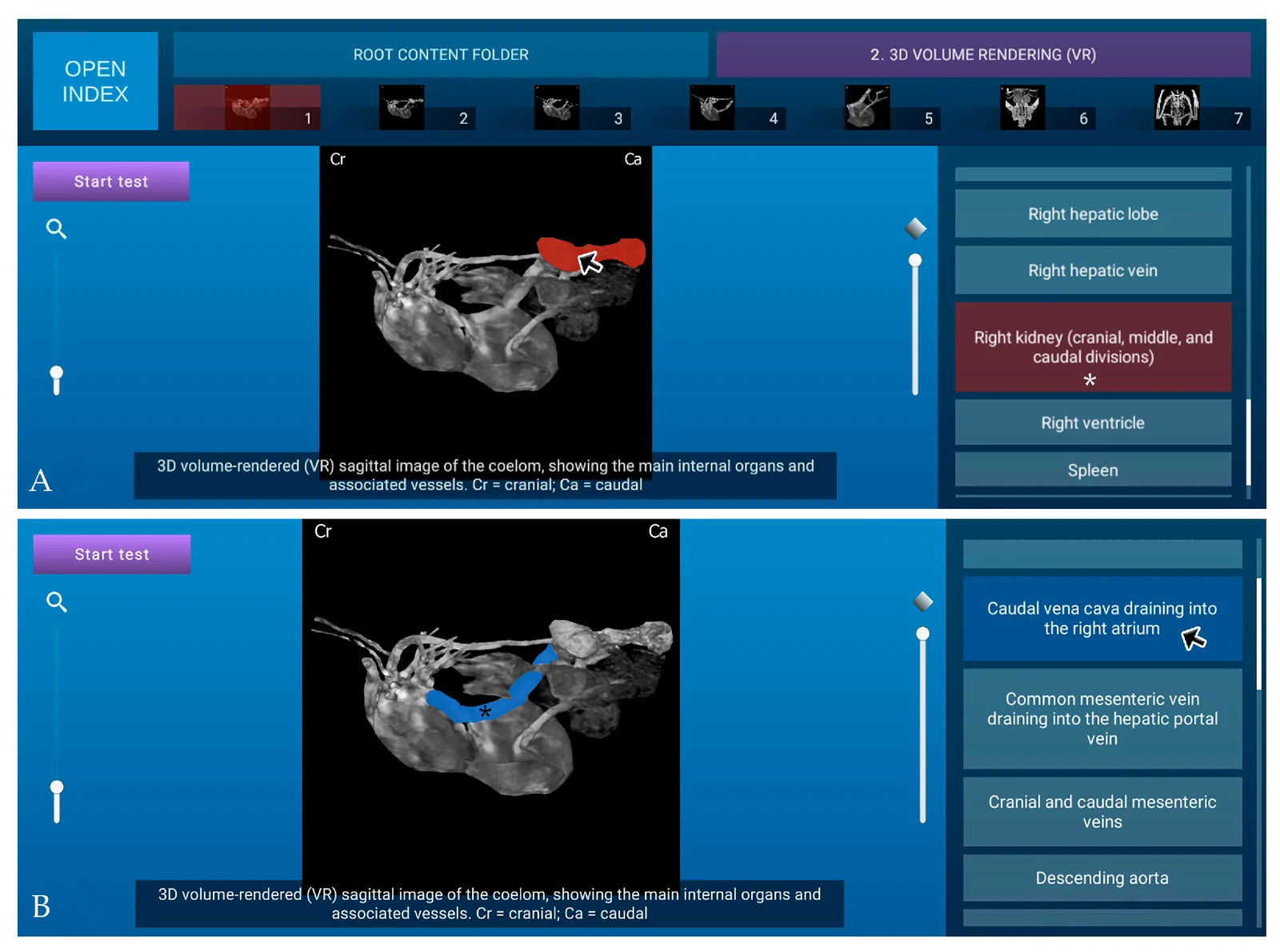
Representative sagittal VR reconstruction (No. 1), viewed from the left lateral aspect. (**A**) Positioning the cursor over a structure highlights it in colour and simultaneously highlights the corresponding label (right kidney; white asterisk). (**B**) Positioning the cursor over a label (caudal vena cava draining into the right atrium) highlights the corresponding anatomical structure (black asterisk).

**Figure 13:**
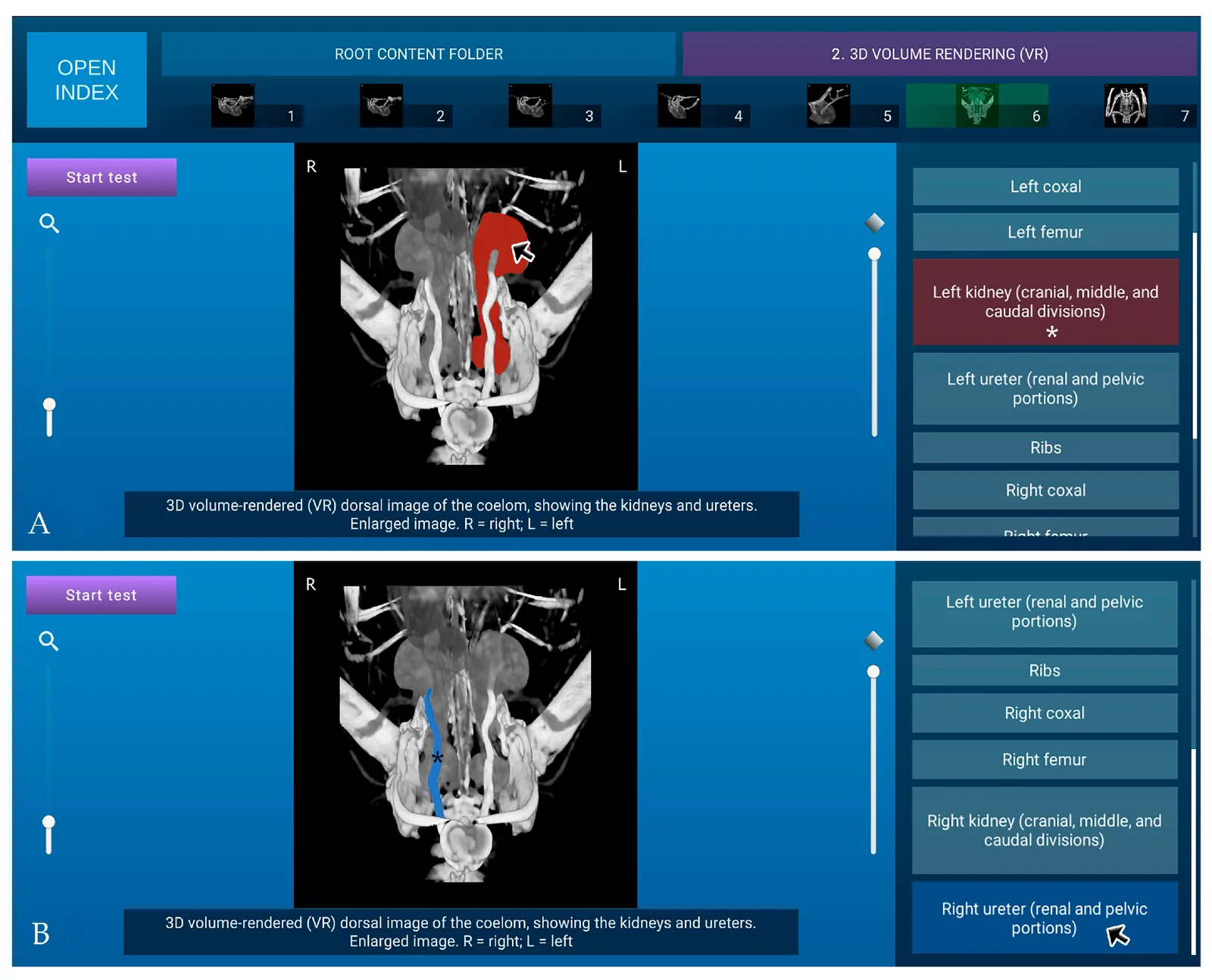
Representative dorsal VR reconstruction (No. 6), viewed from the ventral aspect. (**A**) Positioning the cursor over a structure highlights it in colour and simultaneously highlights the corresponding label (left kidney; white asterisk). (**B**) Positioning the cursor over a label (right ureter) highlights the corresponding anatomical structure (black asterisk).

Overall, the VR reconstructions provided a comprehensive 3D representation of the coelom and were particularly well suited to visualising structures with high or intermediate attenuation. By contrast, low-attenuation structures, including the pulmonary parenchyma and air sacs, were less well visualised. Representative sagittal and dorsal 3D VR reconstructions are shown in Figure 12 and Figure 13, respectively.

Supplementary Table S1 summarizes the main anatomical features identified in the coelom of an adult female bald eagle (*Haliaeetus leucocephalus*) using contrast-enhanced CT imaging.

## 4. Discussion

This preliminary study describes a contrast-enhanced CT-based workflow for the segmentation and anatomical assessment of the coelom in a female bald eagle (*Haliaeetus leucocephalus*). Manual segmentation of the volumetric CT datasets, supported by standardised data organisation and advanced visualisation techniques, enabled detailed and morphologically consistent delineation of the principal avian anatomical structures. This workflow offers a structured framework for organising, visualising, and interpreting anatomical information in a species for which high-resolution cross-sectional imaging data remain limited.

Previous CT-based studies of avian coelom anatomy have focused primarily on descriptive anatomical characterisation [32,35,43]. In contrast, the present study combined contrast-enhanced CT acquisition, systematic manual segmentation, MIP, 3D VR, and interactive visualisation within a unified methodological framework. To the best of our knowledge, this is the first study to integrate these imaging, segmentation, and advanced visualisation techniques for the CT-based anatomical assessment of a female bald eagle (*Haliaeetus leucocephalus*). This integrated approach provides a more comprehensive representation of anatomical relationships within the highly pneumatised avian coelom, enhances spatial interpretation beyond that achievable using conventional static images, and enables interactive exploration of the reconstructed volumetric datasets.

In contrast to conventional CT workflows that rely primarily on transverse images or MPR datasets, the methodology used in the present study combined manual segmentation with MIP and 3D VR reconstructions generated from the original volumetric dataset. This approach enabled more precise delineation of anatomical structures within the complex coelom, where structural superimposition and the close spatial relationships among adjacent soft-tissue structures can hinder interpretation on conventional cross-sectional images. MIP reconstructions can improve the conspicuity of high-attenuation structures, particularly contrast-opacified vessels and osseous elements, whereas 3D VR reconstructions enhance spatial perception and facilitate the contextual interpretation of anatomical relationships [39,40,41].

Collectively, these complementary techniques supported the identification and systematic organisation of anatomical structures during manual segmentation. The source CT dataset and MPR images were used as anatomical references to maintain spatial accuracy and morphological consistency. Interactive visualisation tools also enabled a more comprehensive exploration and interpretation of the reconstructed volumetric datasets. Such tools may have applications in anatomical education, research, and diagnostic imaging.

Contrast-enhanced CT was essential for achieving adequate soft-tissue differentiation within the avian coelom [34]. CT enables the simultaneous visualisation of skeletal, vascular, and soft-tissue structures [32,42,43,44], while the administration of iodinated contrast medium further enhances tissue differentiation [33,34]. This is particularly valuable in regions where close anatomical relationships and structural superimposition limit intrinsic soft-tissue contrast. In the present study, MPR images in the transverse, sagittal, and dorsal planes provided complementary anatomical views and facilitated the assessment of spatial relationships among adjacent structures.

Beyond the imaging characteristics of avian tissues, the specialised organisation of the avian coelom presents additional challenges for cross-sectional anatomical interpretation. Unlike the relatively continuous mammalian abdominal cavity surrounding the viscera, the avian coelom is subdivided into distinct compartments, including the pulmonary pleural cavities, pericardial cavity, hepatic peritoneal compartments, and common intestinal peritoneal cavity [9,10,11]. Furthermore, organs such as the kidneys and adrenal glands occupy a retroperitoneal position and are therefore not enclosed within the peritoneal compartments of the coelom.

Recognition of these anatomical compartments was essential during manual segmentation because the spatial relationships among adjacent structures and the organisation of the coelom directly affected anatomical identification and 3D reconstruction. Incorporating this compartmental framework into the interpretation of the contrast-enhanced CT dataset contributed to a more accurate representation of avian visceral anatomy and supported the development of an anatomically coherent digital resource.

Although automated and semi-automated segmentation techniques continue to advance, manual segmentation remains an accepted approach for generating anatomical ground-truth datasets in non-model species for which validated automated methods are limited or unavailable [45]. In the present study, the use of a standardised segmentation protocol, together with independent assessment by external evaluators, supported the anatomical consistency and reliability of the resulting segmentation. Given the primarily methodological and exploratory nature of the study, formal quantitative validation metrics was beyond its scope.

From a biological perspective, the bald eagle (*Haliaeetus leucocephalus*) exhibits several anatomical features characteristic of birds, including extensive skeletal pneumatisation and a well-developed air-sac system [9,10,11,43]. The close juxtaposition of air-filled structures, bone, and soft tissues produces marked differences in attenuation and can complicate CT image interpretation. From clinical and conservation perspectives, accurate CT-based anatomical reference data may aid the recognition and interpretation of pathological changes associated with infectious, toxicological, traumatic, musculoskeletal, cardiovascular, and emerging conditions affecting bald eagles.

Environmental contaminants, including legacy organochlorine pesticides, lead, mercury, and anticoagulant rodenticides, remain major conservation concerns for bald eagles (*Haliaeetus leucocephalus*) [5,17,18,20,21,22]. A wide range of infectious diseases, including bacterial, parasitic, fungal, and viral infections, also continues to affect both free-ranging birds and individuals undergoing rehabilitation [12,15,19,23,26,28,29]. Musculoskeletal and cardiovascular disorders have likewise been reported in this species, further emphasising the potential value of advanced cross-sectional imaging in the assessment of diverse pathological conditions [13,24,25,27].

However, in cases of toxicosis and certain infectious diseases, CT is likely to provide diagnostic support primarily when structural abnormalities or secondary lesions are present, whereas definitive diagnosis generally requires toxicological, microbiological, parasitological, or molecular testing. Accordingly, accurate CT-based anatomical reference data may improve the detection and interpretation of pathological changes that are not readily identified or fully characterised using conventional radiography [32,33,34,35,36].

Several limitations of this study should be acknowledged. First, the present work should be regarded as a preliminary anatomical and imaging reference rather than as a definitive anatomical atlas. Although the findings demonstrate the feasibility and potential utility of the proposed workflow and provide a valuable anatomical resource, the dataset was not designed to identify and annotate every anatomical structure potentially visible on contrast-enhanced CT of a bird of this size. The anatomical descriptions presented here should therefore not be considered exhaustive.

The accompanying online resource provides an initial framework that can be progressively expanded through the incorporation of additional anatomical structures, more detailed annotations, optimised imaging protocols, complementary anatomical validation, and further imaging datasets. Such developments would enhance its value for anatomical research, education, and clinical imaging applications.

The inclusion of a single female specimen limits the generalisability of the findings and precludes assessment of inter-individual, age-related, and sex-related anatomical variation. Although sexual dimorphism may affect certain coelom structures, the use of a single specimen was considered appropriate for this proof-of-concept study, which aimed to establish an initial anatomical and imaging framework rather than to characterise variation at the population level. Single-specimen CT studies are well established in the veterinary and wildlife imaging literature, particularly in investigations involving raptors and other protected species, for which access to suitable specimens is inherently limited by ethical, legal, and conservation constraints [16,28,37,38].

Additional limitations include the inherent subjectivity of manual segmentation and the intrinsically limited soft tissue contrast within the highly pneumatised avian coelom. To support anatomical plausibility and consistency, the resulting segmentations were independently reviewed by specialists in avian anatomy, diagnostic imaging, and zoological medicine as part of a qualitative quality-assurance process. Nevertheless, this expert review did not constitute formal validation study and did not include quantitative measures of segmentation accuracy or inter-observer agreement.

A reduced dose of iodinated contrast medium was selected to achieve adequate tissue enhancement while minimising potential adverse effects. Although the selected dose provided satisfactory delineation of most anatomical structures, it may have limited the enhancement of some soft tissues and small vascular structures, thereby contributing to the reduced visibility of certain anatomical features.

A further methodological limitation concerns the segmentation of the air sacs and the delineation of their membranous boundaries. Although the air-filled spaces were readily identifiable on lung window CT images, the thin membranes defining their anatomical boundaries were not visualised consistently enough to permit reliable segmentation. Attempting to delineate these structures under such conditions would have increased the risk of interpretative error and reduced the anatomical fidelity of the reconstructed dataset. This limitation most likely reflects the difficulty of resolving these delicate membranes using the imaging protocol applied in the present study. However, the relative contribution of spatial resolution, acquisition parameters, and reconstruction settings could not be determined.

Similarly, although the primary bronchi were readily identified, the intrapulmonary bronchial tree could not be consistently differentiated into the recognised groups of secondary bronchi throughout the lungs. These structures were therefore neither segmented individually nor described in detail, as attempting to do so might have compromised the anatomical accuracy of the reconstructed dataset.

Clinically relevant peripheral nerves, including the sacral and pudendal plexuses, were also specifically assessed during image interpretation but could not be reliably identified on the CT images. Despite their anatomical and clinical importance, these neural structures could not be distinguished from the surrounding soft tissues, most likely because of their small calibre and the limited soft-tissue contrast and spatial resolution achieved with the CT protocol used in this study. Consequently, the peripheral nervous system was not included in the anatomical description. Future studies using higher-resolution imaging, optimised contrast-enhanced CT protocols, or magnetic resonance imaging may permit a more comprehensive assessment of these neural structures.

Certain anatomical structures, including the pancreas, could not be reliably visualised despite the administration of iodinated contrast medium. Pancreatic conspicuity was likely limited by the dimensions and morphology of the organ, its close anatomical association with the duodenal loop, and several technical factors, including the selected contrast-enhancement phase, the spatial resolution of the CT dataset, and the acquisition and reconstruction parameters used. However, the relative contributions of anatomical and technical factors could not be determined.

Individual intestinal segments could not be consistently differentiated because of their comparable attenuation characteristics and limited luminal conspicuity. The left ovary was only partially identified, whereas the right ovary was not visualised. This finding is consistent with the marked reproductive asymmetry of female birds, in which the left ovary is typically functional, and the right gonad undergoes regression [9,10,56]. Overall, these limitations most likely resulted from a combination of inherent anatomical complexity, limited intrinsic CT soft-tissue contrast, the spatial resolution of the dataset, and the conservative contrast administration protocol used in this study. Nevertheless, the possible influence of additional methodological factors cannot be excluded.

Future studies should include larger sample sizes comprising specimens of both sexes to enable comparative anatomical analyses and assessment of inter-individual variation. Optimised contrast-administration protocols, including multiphase contrast-enhanced CT, together with higher spatial resolution acquisitions, should also be investigated to improve tissue enhancement and facilitate the identification of structures that remain difficult to visualise. In addition, formal validation protocols involving multiple observers, quantitative measures of segmentation accuracy, and assessment of interobserver agreement would strengthen the reproducibility and anatomical reliability of the segmented datasets.

Semi-automated and artificial intelligence-assisted segmentation methods may further improve efficiency, reproducibility, and scalability, as suggested by recent studies involving avian imaging and CT reconstruction [33,44]. However, such methods would require appropriate training and validation before their wider application. Collectively, these advances could support more extensive comparative anatomical investigations while ensuring continued compliance with the ethical, legal, and conservation requirements associated with protected raptor species [6,7].

Despite these limitations, the proposed segmentation workflow provides a reproducible framework for the CT-based anatomical characterisation of the coelom in a female bald eagle (*Haliaeetus leucocephalus*) and offers a valuable preliminary reference for future imaging, educational, clinical, and conservation applications. The integration of contrast-enhanced CT, manual layer-based segmentation, and interactive anatomical visualisation supports a more detailed understanding of avian coelom anatomy and may facilitate the development of comparable digital resources for other wildlife species. The resulting dataset may also serve as a preliminary reference for future anatomical studies and imaging-based conservation initiatives.

## 5. Conclusions

This preliminary study demonstrated the feasibility of combining contrast-enhanced CT, manual segmentation, and advanced visualisation techniques to generate a structured CT-based anatomical resource of the coelom in a female bald eagle (*Haliaeetus leucocephalus*). The proposed workflow enabled the systematic identification and 3D visualisation of a broad range of coelom structures and provided an organised framework for the interpretation of avian cross-sectional anatomy. Although some anatomical structures could not be reliably identified because of the inherent limitations of conventional CT and the imaging protocol used, the resulting dataset provides a valuable preliminary reference that may be further developed through optimised imaging protocols, complementary imaging modalities, additional anatomical annotations, larger sample sizes, and formal validation. This methodological approach may support veterinary education, diagnostic imaging, and comparative anatomical research, and may facilitate the development of similar CT-based anatomical resources for other avian species.

## Data Availability

The contrast-enhanced CT imaging data are freely available through an interactive visualisation platform at: https://coelomiccavitybaldeagle.ulpgc.es (accessed on 30 July 2026).

## Supplementary Materials

The following supporting information can be downloaded at: https://www.mdpi.com/article/10.3390/ani16162546/s1, Table S1. Distinctive anatomical features of the coelom structures of an adult female bald eagle (*Haliaeetus leucocephalus*) identified on CT in the present study.

## Abbreviations

The following abbreviations are used in this manuscript:

API	Application programming interface
CT	Computed tomography
DDT	Dichlorodiphenyltrichloroethane
DICOM	Digital imaging and communications in medicine
FTP	File Transfer Protocol
HU	Hounsfield unit
HTML5	HyperText markup language 5
JSON	JavaScript object notation
MIP	Maximum intensity projection
MPR	Multiplanar reconstruction
URL	Uniform resource locator
VR	Volume rendering
WebGL	Web graphics library
3D	Three-dimensional

## References

[1] Buehler D.A. (2000). Bald Eagle (*Haliaeetus leucocephalus*). Birds of North America Online.

[2] U.S. Fish and Wildlife Service (2007). Bald Eagle (Haliaeetus leucocephalus) Recovery Plan.

[3] Elliott K.H., Elliott J.E., Wilson L.K., Jones I., Stenerson K. (2011). Density dependence in the survival and reproduction of bald eagles: Linkages to chum salmon. J. Wildl. Manag..

[4] Buehler D.A., Rodewald P.G., Mlodinow S.G. (2022). Bald Eagle *(Haliaeetus leucocephalus)*, version 2.0. Birds of the World.

[5] Grier J.W. (1982). Ban of DDT and subsequent recovery of bald eagles. Science.

[6] BirdLife International (2024). *Haliaeetus* *leucocephalus*. The IUCN Red List of Threatened Species 2024: e.T22695144A264598530.

[7] U.S. Fish and Wildlife Service Bald Eagle (*Haliaeetus leucocephalus*) Species Information. https://www.fws.gov.

[8] Goulet R., Bird D.M., Hancock D. (2021). Aspects of the ecology of urban-nesting bald eagles (*Haliaeetus leucocephalus*) in south coastal British Columbia. J. Raptor Res..

[9] King A.S., McLelland J. (1984). Birds: Their Structure and Function.

[10] König H.E., Korbel R., Liebich H.-G. (2016). Avian Anatomy: Textbook and Colour Atlas.

[11] Taylor W.M. (2016). Pleura, pericardium, and peritoneum: The coelomic cavities of birds and their relationship to the lung–air sac system. Current Therapy in Avian Medicine and Surgery.

[12] Heatley J.J., Mitchell M.M., Roy A., Cho D.Y., Williams D.L., Tully T.N. (2007). Disseminated mycobacteriosis in a bald eagle (*Haliaeetus leucocephalus*). J. Avian Med. Surg..

[13] Sánchez-Migallon Guzman D.S., Bubenik L.J., Lauer S.K., Vasanjee S., Mitchell M.A. (2007). Repair of a coracoid luxation and a tibiotarsal fracture in a bald eagle (*Haliaeetus leucocephalus*). J. Avian Med. Surg..

[14] Stauber E., Holmes S., DeGhetto D.L., Finch N. (2007). Magnetic resonance imaging is superior to radiography in evaluating spinal cord trauma in three bald eagles (*Haliaeetus leucocephalus*). J. Avian Med. Surg..

[15] McAllister C.T., Duszynski D.W., McKown R.D. (2013). A new species of *Caryospora* (Apicomplexa: Eimeriidae) from the bald eagle, *Haliaeetus leucocephalus* (Accipitriformes: Accipitridae), from Kansas. J. Parasitol..

[16] Fernandes A.F., Fenton H., Martinson S., Desmarchelier M., Ferrell S.T. (2014). Absolute polycythemia in a bald eagle (*Haliaeetus leucocephalus*). J. Zoo Wildl. Med..

[17] Eleftheriou A., Murphy L., Welte S. (2017). Evaluation of lead and mercury prevalence in bald eagles (*Haliaeetus leucocephalus*) from the Mid-Atlantic United States. J. Zoo Wildl. Med..

[18] Martin P.A., Hughes K.D., Campbell G.D., Shutt J.L. (2018). Metals and organohalogen contaminants in bald eagles (*Haliaeetus leucocephalus*) from Ontario, 1991–2008. Arch. Environ. Contam. Toxicol..

[19] Goldberg T.L., Sibley S.D., Pinkerton M.E., Dunn C.D., Long L.J., White L.C., Strom S.M. (2019). Multidecade mortality and a homolog of hepatitis c virus in bald eagles (*Haliaeetus leucocephalus*), the national bird of the USA. Sci. Rep..

[20] Manning L.K., Wünschmann A., Armién A.G., Willette M., MacAulay K., Bender J.B., Buchweitz J.P., Redig P. (2019). Lead intoxication in free-ranging bald eagles (*Haliaeetus leucocephalus*). Vet. Pathol..

[21] DeSorbo C.R., Burgess N.M., Nye P.E., Loukmas J.J., Brant H.A., Burton M.E.H., Persico C.P., Evers D.C. (2020). Bald eagle mercury exposure varies with region and site elevation in New York, USA. Ecotoxicology.

[22] Niedringhaus K.D., Nemeth N.M., Gibbs S., Zimmerman J., Shender L., Slankard K., Fenton H., Charlie B., Dalton M.F., Elsmo E.J. (2021). Anticoagulant rodenticide exposure and toxicosis in bald eagles (*Haliaeetus leucocephalus*) and golden eagles (*Aquila chrysaetos*) in the United States. PLoS ONE.

[23] Nemeth N.M., Ruder M.G., Poulson R.L., Sargent R., Breeding S., Evans M.N., Zimmerman J., Hardman R., Cunningham M., Gibbs S. (2023). Bald eagle mortality and nest failure due to clade 2.3.4.4 highly pathogenic H5N1 influenza a virus. Sci. Rep..

[24] Vuong K.S., Jones M., Craig L.E. (2023). Postmortem evaluation of cardiac valvular disease in bald eagles (*Haliaeetus leucocephalus*) and a golden eagle (*Aquila chrysaetos*). J. Avian Med. Surg..

[25] Legler M., von Dörnberg K., Wohlsein P., Fehr M. (2024). Heterotopic ossification of the adductor muscles in bald eagles (*Haliaeetus leucocephalus*). Vet. Sci..

[26] Gould C.V., Staples J.E., Guagliardo S.A.J., Martin S.W., Lyons S., Hills S.L., Nett R.J., Petersen L.R. (2025). West Nile virus: A review. JAMA.

[27] Scott S., Aherne M., Georges C., Adin D.B., Wellehan J.F.X. (2025). Takotsubo cardiomyopathy-like transient systolic dysfunction in two bald eagles (*Haliaeetus leucocephalus*). J. Avian Med. Surg..

[28] Perdrizet U.G., Lockerbie B., Bollinger T.K. (2026). *Myxidium anatidum* in a bald eagle *Haliaeetus leucocephalus* from western Canada. J. Wildl. Dis..

[29] Stilz C.R., Kunkel M.R., Keel M.K., Fenton H., Weyna A.A.W., Niedringhaus K.D., Andreasen V.A., McKinney A.S., Maboni G., Nemeth N.M. (2025). Aspergillosis in 41 wild bird species in the eastern United States: A 22-year retrospective review. J. Vet. Diagn. Investig..

[30] Gumpenberger M., Henninger W. (2001). The use of computed tomography in avian and reptile medicine. Semin. Avian Exot. Pet. Med..

[31] Vilaplana Grosso F. (2019). Orthopedic diagnostic imaging in exotic pets. Vet. Clin. N. Am. Exot. Anim. Pract..

[32] Jaber J.R., Fumero-Hernández M., Corbera J.A., Morales I., Amador M., Ramírez Zarzosa G., Encinoso M. (2023). Cross-sectional anatomy and computed tomography of the coelomic cavity in juvenile Atlantic puffins (Aves, *Alcidae*, *Fratercula arctica*). Animals.

[33] Lautenschlager S., Bright J.A., Rayfield E.J. (2014). Digital dissection—Using contrast-enhanced computed tomography scanning to elucidate hard- and soft-tissue anatomy in the Common Buzzard *Buteo buteo*. J. Anat..

[34] Hein R.F., Kiefer I., Pees M. (2022). A spectral computed tomography contrast study: Demonstration of the avian cardiovascular anatomy and function. Vet. Clin. N. Am. Exot. Anim. Pract..

[35] Orosz S.E., Toal R.L. (1992). Tomographic anatomy of the golden eagle (*Aquila chrysaetos*). J. Zoo Wildl. Med..

[36] Krautwald-Junghanns M.E., Kostka V.M., Dörsch B. (1998). Comparative studies on the diagnostic value of conventional radiography and computed tomography in evaluating the heads of psittacine and raptorial birds. J. Avian Med. Surg..

[37] Wernick M.B., Dennler M., Beckmann K., Schybli M., Albini S., Hoop R.K., Steffen F., Kircher P., Hatt J.M. (2014). Peripheral nerve sheath tumor in a subadult golden eagle (*Aquila chrysaetos*). J. Avian Med. Surg..

[38] Baptista C.S., Monteiro C., Fernandes H., Canadas A., Guardão L., Santos J.C. (2018). Acute intraparenchymal cerebral haemorrhage in an Iberian golden eagle—A case report. BMC Vet. Res..

[39] Maher M.M., Kalra M.K., Sahani D.V., Perumpillichira J.J., Rizzo S., Saini S., Mueller P.R. (2004). Techniques, clinical applications and limitations of 3D reconstruction in CT of the abdomen. Korean J. Radiol..

[40] Kalender W.A. (2011). Computed Tomography: Fundamentals, System Technology, Image Quality, Applications.

[41] Thrall D.E. (2018). Textbook of Veterinary Diagnostic Radiology.

[42] Krautwald-Junghanns M.E., Pees M., Reese S., Tully T.N. (2011). Diagnostic Imaging of Exotic Pets: Birds, Small Mammals, Reptiles.

[43] Veladiano I.A., Banzato T., Bellini L., Montani A., Catania S., Zotti A. (2016). Normal computed tomographic features and reference values for the coelomic cavity in pet parrots. BMC Vet. Res..

[44] Petnehazy O., Csoka A., Fajtai D., Echols S., Donko T. (2024). CT-based 3D reconstruction and basic anatomical analysis of the 3D anatomy of the air sac system in domestic birds. Anat. Histol. Embryol..

[45] Xu Y., Quan R., Xu W., Huang Y., Chen X., Liu F. (2024). Advances in medical image segmentation: A comprehensive review of traditional, deep learning and hybrid approaches. Bioengineering.

[46] Liyanage K.A., Steward C., Moffat B.A., Opie N.L., Rind G.S., John S.E., Ronayne S., May C.N., O’Brien T.J., Milne M.E. (2016). Development and implementation of a corriedale ovine brain atlas for use in atlas-based segmentation. PLoS ONE.

[47] Milne M.E., Steward C., Firestone S.M., Long S.N., O’Brien T.J., Moffat B.A. (2016). Development of representative magnetic resonance imaging–based atlases of the canine brain and evaluation of three methods for atlas-based segmentation. Am. J. Vet. Res..

[48] Li Z., Sutkus L.T., Fil J.E., Senthil P., Lam F., Sutton B.P., Dilger R.N. (2025). PigBET: A 2.5D deep learning segmentation framework for multimodal and longitudinal domestic pig MRI utilizing ImageNet pre-trained encoders. Brain Organoid Syst. Neurosci. J..

[49] Sunico S.K., Hamel C., Styner M., Robertson I.D., Kornegay J.N., Bettini C., Parks J., Wilber K., Smallwood J.E., Thrall D.E. (2012). Two anatomic resources of canine pelvic limb muscles based on CT and MRI. Vet. Radiol. Ultrasound.

[50] Kvam J., Gangsei L.E., Kongsro J., Schistad Solberg A.H. (2018). The use of deep learning to automate the segmentation of the skeleton from CT volumes of pigs. Transl. Anim. Sci..

[51] Trolinger-Meadows K.D., Biedrzycki A.H., He H., Werpy N. (2021). Three-dimensional segmentation and in silico comparison of equine deep digital flexor tendon pathology in horses undergoing repeated MRI examination. Front. Vet. Sci..

[52] Arencibia A., Melián A., Orós J. (2021). Anatomic interactive atlas of the loggerhead sea turtle (*Caretta caretta*) head. Animals.

[53] Park J., Cho H., Ji Y., Lee K., Yoon H. (2024). Detection of spondylosis deformans in thoracolumbar and lumbar lateral X-ray images of dogs using a deep learning network. Front. Vet. Sci..

[54] Lee S., Shimbo G., Yokoyama N., Nakamura K., Togo R., Ogawa T., Haseyama M., Takiguchi M. (2025). Deep-learning-based automatic liver segmentation using computed tomography images in dogs. Front. Vet. Sci..

[55] Arencibia A., Melián A., Orós J. (2026). Anatomic interactive atlas of the loggerhead sea turtle (*Caretta caretta*) coelomic cavity. Animals.

[56] Heidrich A., Schmidt J., Zimmermann J., Saluz H.P. (2013). Automated segmentation and object classification of CT images: Application to in vivo molecular imaging of avian embryos. Int. J. Biomed. Imaging.

[57] Baumel J.J., King A.S., Breazile J.E., Evans H.E., Berge V. (1993). Handbook of Avian Anatomy: Nomina Anatomica Avium.

[58] Lavrenov A. (2026). Konva, Version 10.3.0. https://www.npmjs.com/package/konva/v/10.3.0.

[59] Unity Technologies. Unity 6.4 User Manual. https://docs.unity3d.com/6000.4/Documentation/Manual/UnityManual.html.

