# Supplementary material for: Contrast-Enhanced CT of the Coelom in a Female Bald Eagle (Haliaeetus leucocephalus): Development of a Preliminary CT-Based Anatomical Segmentation Workflow
Source: Animals (Basel). 2026 Aug 14;16(16):2546. doi: 10.3390/ani16162546 (PMC13508918; doi:10.3390/ani16162546)
Supplement: Supplementary file 1 [file animals-16-02546-s001.zip › animals-4411068-supplementary.pdf]

Supplementary table

Table S1. Distinctive anatomical features of the coelom structures of an adult female bald eagle (*Haliaeetus leucocephalus*) identified using contrast-enhanced CT.

ANATOMICAL STRUCTURE	CT SOFT-TISSUE WINDOW	CT LUNG WINDOW	3D VR
**Cortical bone**	Markedly hyperattenuating osseous structures with well-defined cortical margins.	Markedly hyperattenuating osseous structures with sharply delineated cortical margins.	Highly detailed representation of the rigid skeletal framework.
**Bone marrow**	Hypoattenuating relative to cortical bone.	Poorly defined and partially obscured by air filled structures.	Not visualised.
**Muscles**	Soft-tissue structures of moderate attenuation.	Poor contrast relative to the surrounding soft tissues.	Not visualised.
**Heart**	Moderately hyperattenuating with well-defined margins.	Improved visualisation owing to the contrast provided by the adjacent aerated lungs.	Clearly visualised as a central cardiac structure.
**Blood vessels**	Hyperattenuating structures with well-defined margins.	Markedly hyperattenuating branching structures, with high contrast against the lungs and air sacs.	Complex vascular network with clearly defined branching patterns.

**Trachea**	Air-filled tubular structure of very low attenuation, surrounded by a thin soft-tissue wall of intermediate attenuation.	Air-filled tubular structure of very low attenuation with thin, barely perceptible walls.	Not visualised.
**Syrinx**	Structure of soft-tissue attenuation, consistent with its cartilaginous and membranous components.	Air–soft-tissue interface comprising an air-filled lumen of very low attenuation outlined by thin soft-tissue walls.	Not visualised.
**Lungs**	Markedly hypoattenuating parenchyma.	Improved delineation of the pulmonary structures, with heterogeneous attenuation.	Pulmonary parenchyma not visualised.
**Air sacs**	Air-filled cavities of very low attenuation.	Very low-attenuation cavities clearly distinguishable from the pulmonary parenchyma.	Not visualised.
**Oesophagus**	Mildly hyperattenuating tubular structure with well-defined margins.	Narrow, mildly hyperattenuating tubular structure with increased conspicuity.	Tubular structure with limited delineation.
**Glandular stomach (Proventriculus)**	Slender tubular structure with thin walls, uniform contrast enhancement, and clear delineation.	Structure of intermediate attenuation with reduced soft-tissue contrast.	Tubular structure with limited delineation.

**Muscular stomach (Ventriculus)**	Large muscular structure with homogeneous moderate attenuation.	Structure of intermediate attenuation with poor soft-tissue contrast.	Rounded muscular structure with moderate delineation.
**Liver**	Homogeneous, mildly hypoattenuating hepatic parenchyma.	Suboptimal evaluation of the hepatic parenchyma owing to reduced soft-tissue contrast.	Well-defined bilobed organ within the cranial coelom.
**Gallbladder**	Thin-walled, fluid-filled structure of low attenuation.	Poorly delineated owing to suboptimal soft-tissue contrast.	Not identified.
**Spleen**	Small, mildly hyperattenuating, well-circumscribed oval structure.	Poorly defined owing to low soft-tissue contrast.	Small structure adjacent to the stomach.
**Pancreas**	Not visualised.	Not visualised.	Not visualised.
**Intestines**	Thin-walled intestinal loops containing variable amounts of intraluminal gas and fluid.	Limited evaluation of the intestinal loops owing to suboptimal soft-tissue contrast.	Coiled tubular structures occupying the central and caudal regions of the coelom.

Table S1 (Cont.)

ANATOMICAL STRUCTURE	CT SOFT-TISSUE WINDOW	CT LUNG WINDOW	3D VR
**Kidneys**	Paired, lobulated, moderately hyperattenuating structures located retroperitoneally.	Hyperattenuating structures with limited soft-tissue differentiation.	Paired, elongated structures embedded within the synsacrum.
**Adrenal glands**	Small, faintly visible soft-tissue structures located cranial to the kidneys.	Not clearly visualised because of limited soft-tissue contrast.	Not visualised.
**Ureters**	Paired, slender, thin-walled tubular structures of soft-tissue attenuation, variably visualised following contrast administration.	Poorly defined tubular structures.	Paired, slender tubular structures extending from the kidneys to the cloaca.
**Ovaries**	The left ovary appeared as a small, irregular, mildly hyperattenuating structure cranial to the left kidney; the right ovary was not visualised.	Assessment was limited by poor soft-tissue contrast; the right ovary was not visualised.	Not visualised.
**Nerves**	Not visualised.	Not visualised.	Not visualised.
